# Gene expression analysis delineates the potential roles of multiple interferons in systemic lupus erythematosus

**DOI:** 10.1038/s42003-019-0382-x

**Published:** 2019-04-23

**Authors:** Michelle D. Catalina, Prathyusha Bachali, Nicholas S. Geraci, Amrie C. Grammer, Peter E. Lipsky

**Affiliations:** AMPEL BioSolutions LLC and RILITE Research Institute, 250 West Main Street, Suite 300, Charlottesville, VA 22902 USA

**Keywords:** Transcriptomics, Systemic lupus erythematosus, Microarrays, Interferons

## Abstract

A role for interferon (IFN) in systemic lupus erythematosus (SLE) pathogenesis is inferred from the prominent IFN gene signature (IGS), but the major IFN species and its relationship to disease activity are unknown. A bioinformatic approach employing individual IFN species gene signatures to interrogate SLE microarray datasets demonstrates a putative role for numerous IFN species, with prominent expression of IFNB1 and IFNW signatures. In contrast with other SLE-affected organs, the IGS is less prominent in lupus nephritis. SLE patients with active and inactive disease have readily detectable IGS and the IGS changes synchronously with a monocyte signature but not disease activity, and is significantly related to monocyte transcripts. Monocyte over-expression of three times as many IGS transcripts as T and B cells and IGS retention in monocytes, but not T and B cells from inactive SLE patients contribute to the lack of correlation between the IGS and SLE disease activity.

## Abstract (web summary)

Michelle Catalina et al. use a bioinformatic approach to identify interferon genes with potential roles in systemic lupus erythematosus (SLE). They predict an important role for IFNB1 in SLE pathogenesis in skin and synovium, but not SLE renal disease.

## Introduction

A role for interferon (IFN) in the pathogenesis of systemic lupus erythematosus (SLE) has been proposed since early experiments showed elevated IFN activity in SLE patients^1,2^ and the advent of gene expression profiling demonstrated a robust IFN gene signature (IGS) in SLE patient peripheral blood, purified B cells, T cells, monocytes, and affected organs^3–7^. Various IFN responsive genes have been used to define the IGS^8,9^ but little is understood regarding the specific species of IFN underlying the signature. Notably, there is no consensus concerning the association of the IGS with SLE disease activity. Although some disease metrics have been associated with the IGS in small studies^10–14^, longitudinal studies have not shown correlation between the IGS and disease activity^15,16^.

Anecdotal accounts of patients developing SLE-like symptoms after treatment with IFNs have been reported^17–23^ suggesting that IFN might play a role in the induction of SLE. Moreover, standard of care (SOC) drugs used to treat lupus may eliminate the IGS^4,24^. Two anti-IFNA antibodies have been used to treat SLE in Phase II clinical trials but with only modest effects^25,26^. In contrast, a phase 2 trial using the antibody anifrolumab which blocks binding of all type I IFNs to the shared IFN receptor provided clinically meaningful benefit in subjects with SLE and with high IGS scores^27^. These trials raised the important question of whether IFNA is the predominant IFN acting in SLE.

An IGS may be induced by type I or type II IFNs. The human type I IFN locus comprises thirteen *IFNA* genes (*A1*, *A2*, *A4*, *A5*, *A6*, *A7*, *A8*, *A10*, *A13*, *A14*, *A16*, *A17*, and *A21)*, *IFNB1*, *IFNW1*, and *IFNE*^28^. Despite a similarity in structure and common receptor, these IFNs may induce different downstream signaling events^29,30^, although mRNA signatures to distinguish the action of a specific subtype of type I IFN have not been developed or employed to delineate the actions of specific Type 1 IFNs. The type II IFN, IFNG, also induces an IGS through its distinct IFNG receptor and has been shown to be important for pathogenesis in lupus mouse models^31,32^. The role of IFNG in the pathogenesis of human lupus has been inferred largely through in vitro experiments^33–35^.

Efforts to deconvolute the IGS in SLE have previously been attempted by creating three modules of IFN genes (M1.2, M3.4, M5.12) from SLE microarray datasets clustered using a K-means algorithm on the basis of their expression^36^. Some correlation between module 5.12 with SLE flares was noted and characterization of the module using the IFN database, the Interferome^37^, was done in an attempt to classify the species of IFN^38^.

This study uses a systems-level approach of probing multiple, publicly available gene expression datasets from SLE patients with modules of genes derived from reference datasets of the downstream IGS. This study demonstrates the relative contributions of different types of IFN in SLE tissues and cells. Skin and synovium from SLE patients have highly enriched downstream IFN signatures with IFNB1 signatures predominating whereas lupus nephritis kidneys have comparatively lower expression of all downstream IFN signatures. This study further demonstrates that the IGS is readily detectable in patients with inactive disease and does not change synchronously with disease activity. The lack of correlation of the IGS with SLE disease activity is likely related to the retention of the IGS in monocytes from patients with inactive disease making interpretation of the IGS as a measure of disease activity difficult.

## Results

### Type I and Type II IGS are present in SLE cells and tissues

**Fig. 1:**
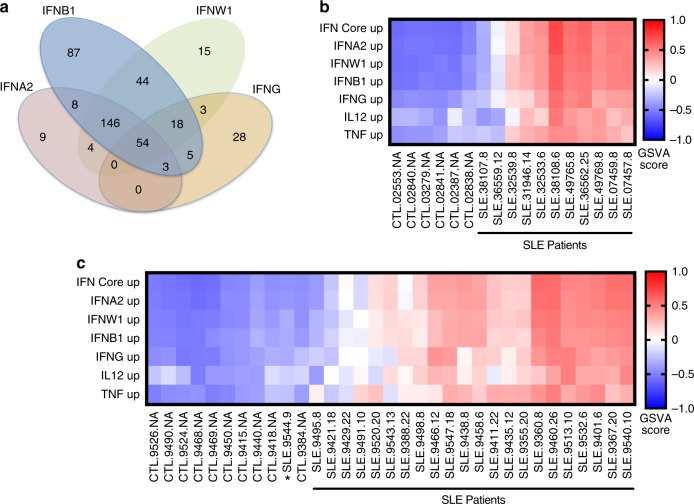
IFN subtype signatures separate SLE patients from controls. **a** Venn Diagram of the overlap of transcripts Induced in human PBMC after 24-h treatment with IFNA2, IFNB1, IFNW1 or IFNG. A 200 gene signature common to the three type I IFNs (IFN Core, 146 + 54) was determined. Gene symbols for the induced transcripts for each IFN are listed in Supplementary Data 2. **b**, **c** GSVA is an unsupervised methodology which calculates enrichment scores between −1 and 1 for groups of genes potentially co-expressed in individual subjects. Because GSVA normalizes the log2 expression data and allows incorporation of healthy control values in the calculation to standardize the enrichment scores, it mitigates against the strong batch effects demonstrated for microarray data^40^ and allows a direct comparison of enrichment scores across multiple datasets. The induced transcripts from IFN or cytokine treatment of PBMC were used as enrichment groups for GSVA analysis of (**b**) SLE patient PBMC (FDA PBMC), or (**c**) SLE whole blood (GSE49454) patients with SLEDAI ≥ 6. Heatmap visualization uses red (enriched signature) for GSVA values above zero and blue (decreased signature) for GSVA values below zero to show differences between SLE patients and controls. SLE patients were considered positive for a signature if their GSVA enrichment score was greater than the average healthy control GSVA enrichment score plus two standard deviations. Most SLE patients displayed prominent type I IFN signatures. **c** Patients SLE.9520, SLE.9491, and SLE.9495 had enriched PBMC-TNF signatures compared to IFN signatures and patient SLE.9544 (*) had no PBMC-IFN signature and grouped with controls

Microarray data derived from publicly available datasets and collaborators was used to define the subtype of IFN in SLE patients (Supplementary Data 1). Genes induced by the in vitro stimulation of normal human PBMC with IFNA2, IFNB1, IFNW1 or IFNG, and as controls the signatures induced by TNF or IL12^39^ were employed as reference modules. Figure 1a depicts a 54 transcript shared type I and type II IGS and a 200 transcript shared type I IGS (IFN Core; Supplementary Data 2). Each IFN also induced a unique IGS (Supplementary Fig. 1). Of note, comparison of these groups of IFN-induced transcripts to the Chiche-Chaussabel^38^ IFN modules previously described demonstrated the transcripts in common were in the shared IFN core signature and thus the three previously described IFN modules did not appear to represent genes induced by specific IFNs (Supplementary Fig. 2).

Gene Set Variation Analysis (GSVA)^40^ using the IFNA2, IFNB1, IFW1, IFNG, TNF, IL12, and the IFN Core signature genes (Supplementary Data 2) were employed to determine the relative enrichment of these signatures in SLE patient and control whole blood (WB) or peripheral blood mononuclear cells (PBMC). Heatmap visualization of GSVA enrichment scores demonstrated patients with highly enriched signatures for IFNA2, IFNB1, IFNW1, IFNG, and the IFN core and that most SLE patients were separated from controls by these signatures. In contrast, GSVA using random groups of genes did not separate SLE patients from controls (Supplementary Data 2; Supplementary Fig. 3). In most SLE patients, the GSVA enrichment scores were the strongest for the type 1 IFNs compared to IFNG, TNF, or IL12. However, some patients had no type I or type II IGS, but did possess a TNF or IL12 signature (Fig. 1b, c).

### Enrichment of the IFNB1 signature in SLE skin and synovium

**Fig. 2:**
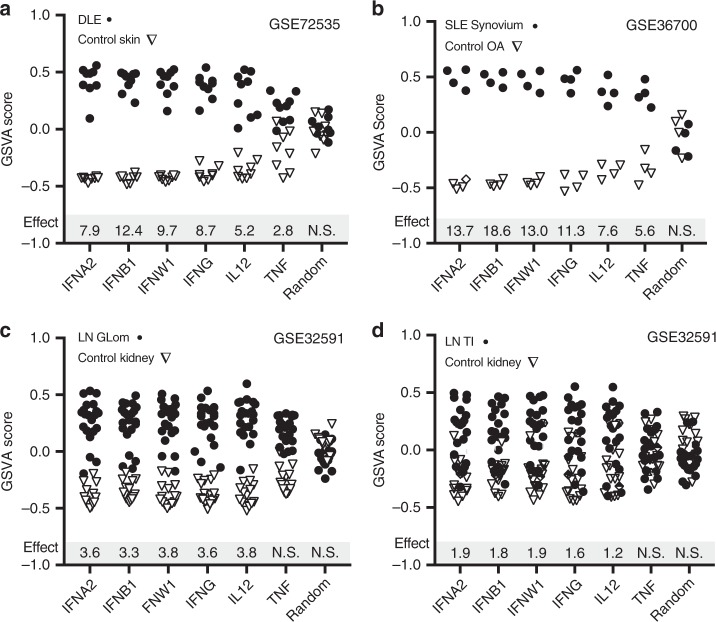
IFNA2, IFNB1, and IFNW1 signatures separate SLE tissues from controls. GSVA enrichment scores using the PBMC IFNA2, IFNB1, IFNW1, IFNG, IL12, or TNF-induced transcripts and a random signature (Random Gr1) (Supplementary Data 2, Supplementary Fig. 3) were calculated for (**a**) discoid lupus erythematosus (DLE) and healthy control skin, (**b**) SLE synovium and osteoarthritis (OA) synovium, (**c**) lupus nephritis (LN) glomerulus (Glom) class III/IV and control kidney Glom and (**d**) LN tubulointerstitium (TI) class III/IV and control kidney TI. Hedge’s G effect size (*Effect*) measures are shown for cytokine signatures significantly enriched in SLE affected tissues compared to control tissues as determined by a *p* value < .05 using the Welch’s *t*-test. N.S. signifies a Welch’s *t*-test value ≥ .05. For LN tissues, recalculation of effect size values without the five IFN negative tissues roughly doubled the effect size values for the type I IFNs: IFNW1 (Glom *g* = 5.5, TI *g* = 3.3), IL12 (Glom *g* = 4.9, TI *g* = 1.9); IFNG (Glom *g* = 5.5, TI *g* = 2.2), IFNB1 (Glom *g* = 6.0, TI *g* = 3.0), IFNA2 (Glom *g* = 6.6, TI *g* = 3.1), but they were still lower than the effect size values calculated for the DLE and SLE synovium

GSVA enrichment scores using the IFN signatures also separated SLE affected organs from controls. Discoid lupus erythematosus (DLE) was significantly separated from control skin by all of the signatures (*p* < .05); IFNB1 had the greatest size (Hedge’s *g* = 12.4) followed by IFNW1 (*g* = 9.7), IFNG (*g* = 8.7), IFNA2 (*g* = 7.9), IL12 (*g* = 5.2), and TNF (*g* = 2.8) (Fig. 2a). In SLE synovium, all six signatures were significantly enriched in SLE patients compared to control osteoarthritis (OA) tissue (*p* < .05) and the effect size was the greatest for the IFNB1 signature (*g* = 18.6), followed by IFNA2 (*g* = 13.7), IFNW1 (*g* = 13), IFNG (*g* = 11.3), IL12 (*g* = 7.6), and TNF (*g* = 5.6)(Fig. 2b). In kidney glomerulus (Glom) and tubulointerstiitum (TI) from SLE patients with Class III/IV lupus nephritis (LN) (Fig. 2c, d), there was no significant TNF enrichment but the other five signatures were significantly enriched in SLE patients (*p* < .05). The effect size calculations were more than 50% less than those calculated for DLE and SLE synovium, and five SLE patient kidney tissues had no IGS: IFNW1 had the highest effect size values for LN Glom (*g* = 3.8) and TI (*g* = 1.9) followed by IL12 (Glom *g* = 3.8; TI *g* = 1.2), IFNG (Glom *g* = 3.6, TI *g* = 1.6), IFNA2 (Glom *g* = 3.6, TI *g* = 1.9), and IFNB1 (Glom *g* = 3.3, TI *g* = 1.8).

**Fig. 3:**
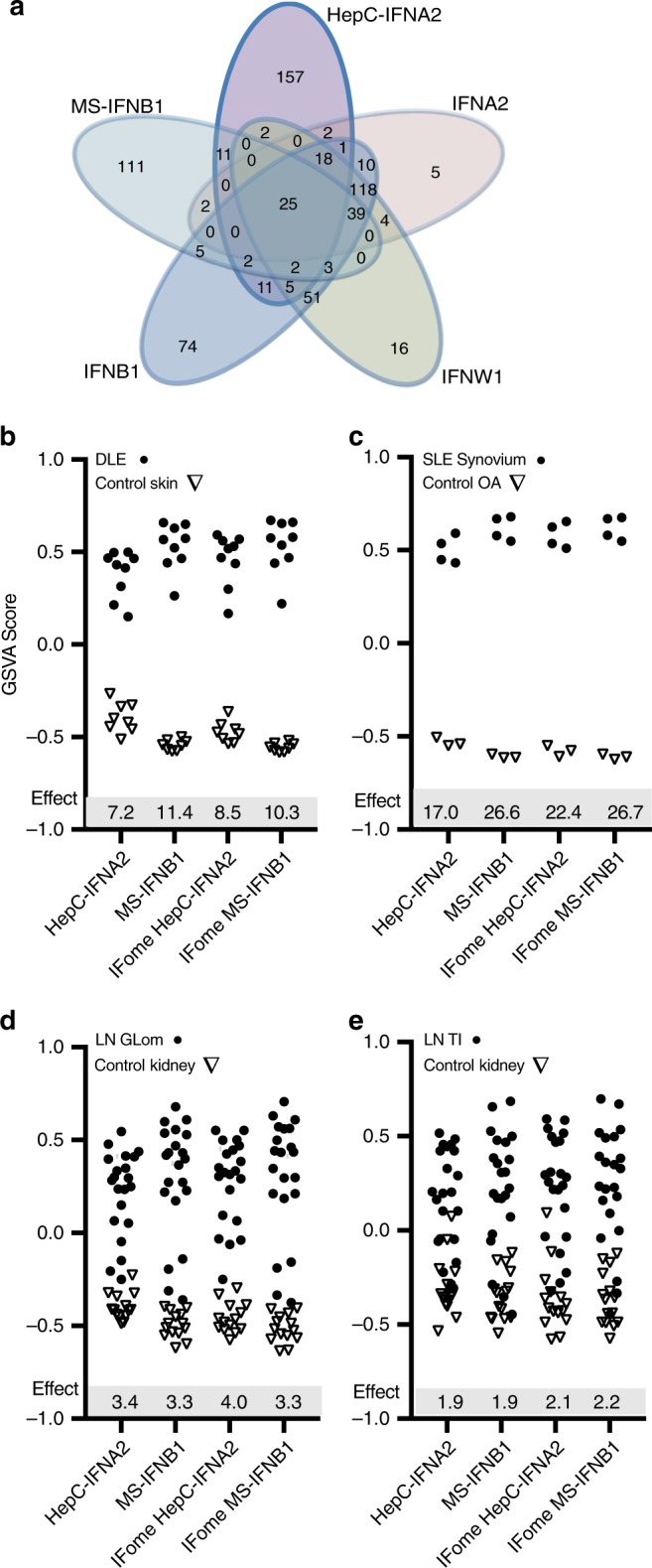
IFN signatures induced in IFNB1 treated MS patients and IFNA2 treated Hepatitis C patients separate SLE patients from controls. The IFNB1 signature was taken from the differential gene expression analysis of WB from 58 multiple sclerosis patients treated with recombinant IFNB1 for one to two years and compared to 62 multiple sclerosis patients not treated with IFNB1 (MS-IFNB1)^41^. The IFNA2 signature was taken from the differential gene expression analysis of PBMC from four hepatitis C patients treated with IFNA2 for 6 h and compared to the same patients before IFNA2 administration (HepC-IFNA2)^42^. **a** Venn diagram overlap of the MS-IFNB1 and HepC-IFNA2 signatures with the PBMC-derived IFNA2, IFNB1 and IFNW1 signatures demonstrated large numbers of unique transcripts for the MS-IFNB1 (111) and HepC-IFNA2 (157) signatures and raised the question of whether the transcripts were indeed directly induced by IFN. The Interferome^37^ was used to determine whether the transcripts were IFN inducible and 87.5% of the induced MS-IFNB1 and 56% of the induced HepC-IFNA2 transcripts were identified as type I IFN genes (Supplementary Data 3). **b**–**e** GSVA using the increased transcripts of MS-IFNB1, HepC-IFNA2 and the transcripts from either signature restricted to only genes listed on the Interferome (Ifome; www.interferome.org)^37^ (Supplementary Data 3) for (**b**) DLE and control skin, (**c**) SLE Synovium and Osteoarthritis (OA), (**d**) Lupus nephritis (LN) Glomerulus (Glom) Class III/IV and control kidney Glom, and (**e**) LN tubulointerstitium (TI) Class III/IV and control kidney TI. Hedge’s G effect size measures (*Effect*) are shown for IFN signatures significantly enriched in SLE affected tissues compared to control tissues as determined by a *p* value < .05 using the Welch’s *t*-test. For LN tissues, removal of the five IFN negative SLE tissues doubled the effect size values for HepC-IFNA2 (Glom *g* = 6.8, TI *g* = 3.1) and MS-IFNB1 (Glom *g* = 7.7, TI *g* = 3.2)

Reference datasets for the IFNB1 signature from multiple sclerosis (MS) patients chronically treated with recombinant IFNB1 compared to untreated MS patients^41^ (MS-IFNB1) and the IFNA2 signature derived from PBMC of hepatitis C patients treated with IFNA2^42^ (HepC-IFNA2) were employed to interrogate the nature of the SLE IGS in greater detail. GSVA was carried out on the four SLE-affected tissues using four signatures: MS-IFNB1, HepC-IFNA2 and each of these signatures with only Interferome database confirmed transcripts (IFome)^37^. All signatures were significantly enriched in all four SLE affected tissues compared to control tissues (*p* < .05). Similar to the pattern seen with the PBMC-derived signatures, the MS-IFNB1 signature had greater effect sizes for both DLE (*g* = 11.4) and synovium (*g* = 26.6) compared to the HepC-IFNA2 values for DLE (*g* = 7.2) and synovium (*g* = 17). Removal of the transcripts not listed in the Interferome did not change the effect size values for the MS-IFNB1 signature but increased the HepC-IFNA2 signature for both DLE and synovium (Fig. 3b, c). Similar to the results determined using PBMC-derived IFN signatures in LN Glom and TI, five patient tissues had no IGS and the calculated effect sizes for HepC-IFNA2 (Glom *g* = 3.4, TI *g* = 1.9) and MS-IFNB1 (Glom *g* = 3.3, TI *g* = 1.9) were lower than for DLE and synovium (Fig. 3d, e). Overall, IFNA2, -IFNB1, -IFNW1, -IFNG, MS-IFNB1, and HepC-IFNA2 downstream signatures were all significantly discriminatory (*p* < .05, two-way ANOVA) between SLE and controls, whereas the MS-IFNB1 signature in the DLE and synovium significantly discriminated (*p* < .05; two-way ANOVA) between SLE, the other IFN signatures and controls (Supplementary Data 4).

**Fig. 4:**
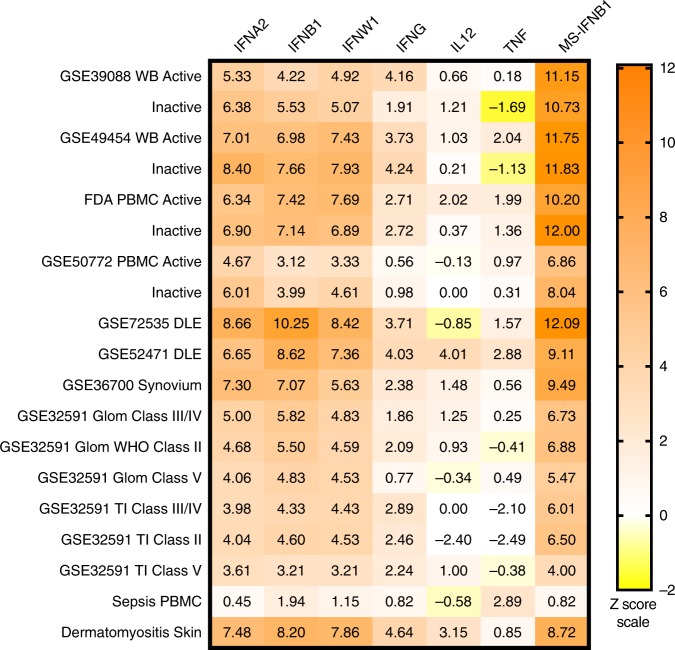
Confirmation of a strong IFNB1 signature in cells and tissues from SLE patients. Z scores were calculated using the differential gene expression results from human PBMC treated with IFNA2, IFNB1, IFNW1, IFNG, IL12, or TNF^39^, Multiple Sclerosis patients treated with IFNB1 (MS-IFNB1)^41^, sepsis PBMC^43^ (control) and dermatomyositis skin^44^ (control) for SLE WB, PBMC and affected tissues (Supplementary Data 5). Z scores > 2 are considered significant. WB and PBMC datasets from active (SLE disease activity index (SLEDAI) ≥ 6) and inactive (SLEDAI < 6) SLE patients were divided and compared to the same controls separately before Z scores were calculated

An orthogonal approach was taken by calculating Z scores using both increased and decreased transcripts from PBMC-derived IFN^39^ or the MS-IFNB1^41^ signatures to determine the most likely IFN active in SLE patient WB, PBMC, and affected tissues. As controls, a sepsis microarray dataset^43^ and a dermatomyositis microarray dataset^44^ were included in this analysis because these conditions have well described roles for either TNF^45^ or IFNB1^46^. Figure 4 (Supplementary Data 5) demonstrated Z scores greater than six using the MS-IFNB1 signature for all SLE WB, PBMC, and SLE affected tissue datasets establishing both a high overlap and shared directionality of transcripts. The Z score for the control sepsis dataset using the MS-IFNB1 signature was not significant (*Z* = .82), whereas the control dermatomyositis dataset was highly significant (*Z* = 8.72). Confirmation of the high degree of overlap between the MS-IFNB1 signature and the SLE datasets was demonstrated by the significant correlation (*p* < .0001) by linear regression to SLE WB, PBMC, and DLE datasets with coefficient of determination (*r*^2^) values of 0.51–0.65 (Supplementary Fig. 4). In addition, both the increased and decreased transcripts for the MS-IFNB1 signature separated SLE cells and tissues from controls (Supplementary Fig. 5). The MS-IFNB1 Z scores were higher than the PBMC-derived Z scores, but IFNW1, IFNA2, and IFNB1 were still highly significant (*Z* > 3) for all SLE WB, PBMC, and affected tissues, the control dermatomyositis dataset, but not the sepsis dataset. Similar to the results for GSVA enrichment, much higher scores were noted for the type I IGS in DLE and SLE synovium compared to LN Glom and TI. IFNG also had significant Z scores for all SLE affected tissues, but generally several standard deviations (SD) lower than the type I IFN scores. TNF had significant Z scores for one DLE and one WB SLE dataset; the highest Z score for TNF was obtained with the control sepsis dataset. Interestingly, Z scores were similar for SLE WB and PBMC datasets derived from active (SLE Disease Activity Index (SLEDAI) ≥ 6) and inactive (SLEDAI < 6) patients.

The alternative IFNB1 downstream signaling gene expression signature induced by the unique ability of IFNB1 to bind the IFN-Alpha/Beta Receptor 1 was taken from a published experiment in which IFN-Alpha/Beta Receptor 2 deficient mouse cells were treated with IFNB1^30^. The increased transcripts (Supplementary Data 2) were used as a GSVA module to determine whether there was alternative IFNB1 signaling in SLE affected tissues. GSVA enrichment scores for SLE patients showed low enrichment in SLE synovium (*p* = .02, *g* = 2.45), and LN Glom (*p* = .01, *g* = .95) and no enrichment in DLE or LN TI (Supplementary Fig. 6). Taken together, both GSVA and Z score calculations suggest canonical, but not alternative IFNB1 downstream signatures are strongly enriched in SLE PBMC, WB, skin and synovium, and this downstream signature along with the IFNA2 and IFNW1 signatures are less prominent in LN.

### SLE patients with low disease activity exhibit the IGS

**Fig. 5:**
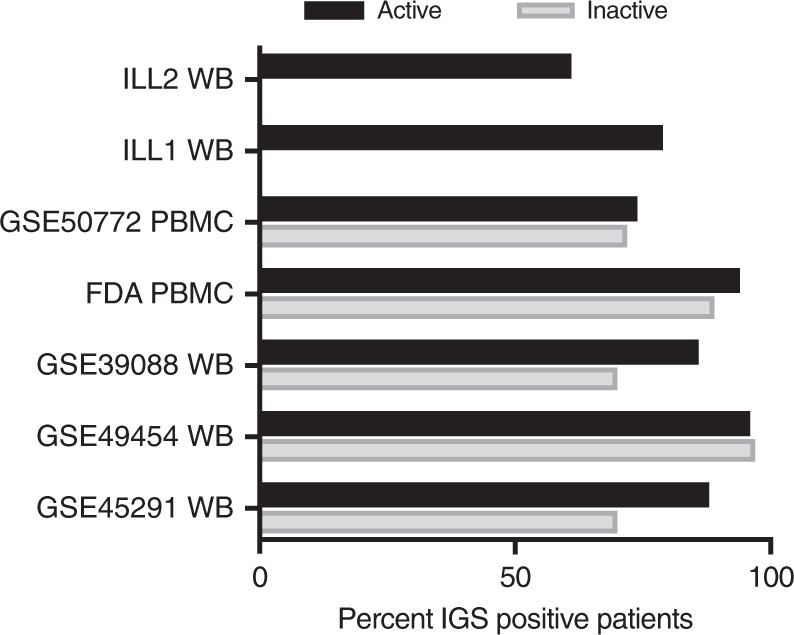
The IGS is readily detected in active and inactive SLE patients. Seven SLE microarray datasets were divided into active SLE patients with SLEDAI ≥ 6 (1722 patients total) or inactive SLE patients with SLEDAI < 6 (315 patients total) (Supplementary Data 6). GSVA enrichment scores were calculated for each patient using the IFN Core signature (Supplementary Data 7 lists results from IFNA2, IFNB1, IFNW1, MS-IFNB1, and HepC-IFNA2 signatures). IFN core signature positive patients had GSVA enrichment scores greater than 2 SD higher than the average of the healthy control GSVA enrichment scores

The similar Z score calculations in active and inactive SLE WB and PBMC (Fig. 4) suggested the IGS was expressed equivalently in active and inactive SLE patients. In order to determine the relationship between the IGS and SLE disease severity, five SLE WB and two SLE PBMC datasets were separated into active (SLEDAI ≥ 6) and inactive (SLEDAI < 6) patients (Supplementary Data 6). A mean of 73% of active SLE patients and a mean of 66% of inactive SLE patients expressed the IFN core signature (Fig. 5). The IFNA2, IFNB1, IFNW1, MS-IFNB1, and HepC-IFNA2 signatures yielded similar results (Supplementary Data 7). To further assess the relationship between the IGS and SLE disease activity, Weighted Gene Coexpression Network Analysis (WGCNA) was carried out on four WB and two PBMC SLE datasets and each dataset yielded one module comprising IGS genes. Pearson correlation of the IFN module eigengene to the presence of SLE disease was significant (*p* < .0005) and positive for all datasets (range of *r* = 0.16 to 0.79), but the magnitude of the correlation to disease activity measured by SLEDAI was low and variable (range of *r* = −0.49 to 0.37) even though some of the relationships to SLEDAI were significant (*p* < .05, Supplementary Data 8).

### The IGS varies over time with changes in cell populations

**Fig. 6:**
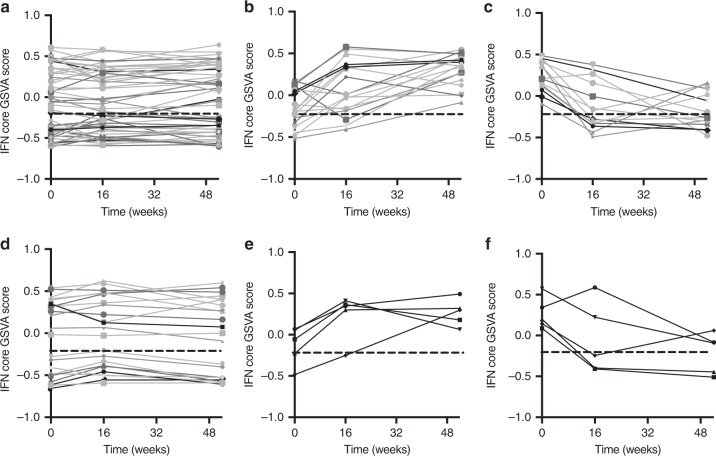
SLE patients may lose or gain the IGS over time. An Ftest differential expression analysis of SLE patients on standard of care treatment at zero, 16 and 52 weeks from SLE time course datasets GSE88885 and GSE88886 was carried out and GSVA enrichment scores were calculated using the IFN core signature. The straight dashed line represents the average IFN core GSVA score plus two standard deviations for the healthy controls and the lines represent SLE patients. Changes in the IGS score of greater than 0.2 SD were considered significant (Supplementary Data 9, 10). For the GSE88885 SLE dataset (**a**) 54 SLE patients had minimal changes in their IGS, (**b**) 18 SLE patients changed from negative to positive score, and (**c**) 14 SLE patients changed from positive to negative enrichment score. For the GSE88886 SLE dataset, (**d**) 23 SLE patients had minimal changes in their IFN core GSVA enrichment score, (**e**) five SLE patients changed from negative to positive, and (**f**) five changed from positive to negative IGS enrichment score

Time course experiments were analyzed to determine whether SLE patients gain or lose the IGS over time. IFN GSVA scores were calculated for SLE patients on SOC treatment at three time points: baseline, 16 weeks and 52 weeks^47^. For the GSE88885 dataset, 60% of subjects expressed an IFN core signature at baseline and 62% (53 patients) had only non-significant changes (SD < 0.2) whereas 38% (33 patients) had significant changes in their IFN core enrichment scores (SD > 0.2) (Fig. 6a**;** Supplementary Data 9). Eighteen patients went from a negative to a positive IGS (Fig. 6b) and 15 patients went from a positive to a negative IGS (Fig. 6c). In the GSE88886 time course dataset, similar changes were noted; 64% of subjects had an IFN core signature at baseline and 70% (23 patients) had only non-significant changes (SD < 0.2) whereas 30% (10 patients) had significant (SD > 0.2) changes in their IFN core GSVA scores (Fig. 6d, Supplementary Data 10). Five patients went from a positive to a negative IGS and five patients went from a negative to positive IGS (Fig. 6e, f). The IFNA2, IFNB1, IFNW1, MS-IFNB1, and HepC-IFNA2 signatures showed similar patterns of change (Supplementary Data 9, 10).

**Fig. 7:**
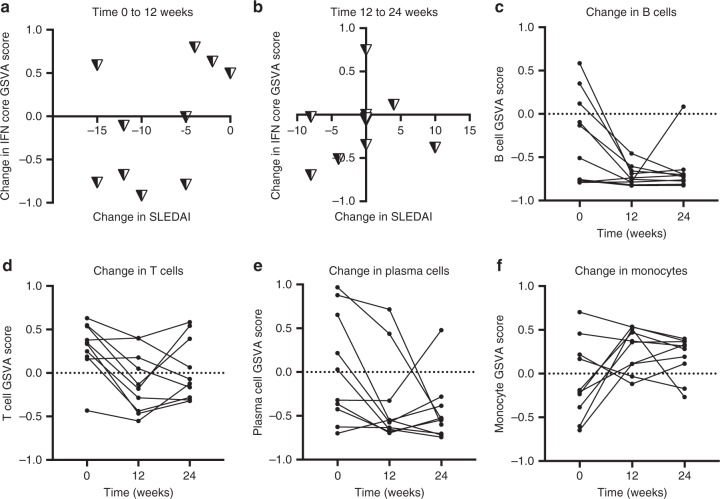
The IGS and SLEDAI do not change synchronously. Ten SLE patients with lupus nephritis and SLEDAI > 8 (GSE72747) and healthy controls (*n* = 46) from GSE39088 had Ftest differential expression analysis using time zero, 12 and 24 week whole blood samples. Treatment with high dose immunosuppressive was begun after time zero and continued for 12 weeks; at 12 weeks all patients were switched to lower dose/maintenance therapy. **a**, **b** Graphs show the change in SLEDAI versus the change in the IFN core signature GSVA enrichment score. **c**–**f** GSVA enrichment signatures corresponding to B cells, T cells, plasma cells, and monocyte cell surface (monocytes, Supplementary Data 12) were determined at each time-point and most patients had standard deviations >.2 between their zero and 12 week time-points. One-way ANOVA *p* values were <.05 for comparison of mean GSVA enrichment scores for B cells, T cells and Monocytes between time zero and 12 weeks. Tukey’s multiple comparison test between time zero and 12 weeks showed significant differences in mean GSVA enrichment scores for B cells (*p* = .02), T cells (*p* = .03) and monocytes (*p* = .05) but not plasma cells

To understand the relationship between the IGS and SLEDAI over time, analysis was carried out on a time course microarray experiment of WB from ten SLE patients with active LN. Samples were taken before therapy (*t* = 0), 12 weeks after treatment with high-dose immunosuppressives (*t* = 12), and after 12 more weeks of moderate-dose to low-dose immunosuppressive therapy (*t* = 24). Nine out of ten patients had changes in their IFN core GSVA scores by 24 weeks (SD > .26; range: .26–.54) (Supplementary Data 11). Figure 7 shows the change in SLEDAI versus the change in the IFN core GSVA score between 0 to 12 weeks (Fig. 7a) and 12 to 24 weeks (Fig. 7b); the other IGS gave similar results and are shown in Supplementary Fig. 7. No consistent pattern for the change in SLEDAI versus the change in IGS was detected. GSVA enrichment scores using gene signatures for T cells, B cells, and monocytes (generated as described in the methods) showed significant changes between 0 and 12 weeks (ANOVA *p* < .05) and there was a relative depletion of plasma cells that was not significant (Fig. 7c–f, Supplementary Data 11, 12). These results demonstrated that at least 30% of SLE patients on SOC may have a significant change in their IGS over time, and that changes in cell populations because of immunosuppressive therapy may significantly affect the IFN signature, but there is no association between the IGS and the SLEDAI.

### The IGS correlates with a monocyte signature

**Fig. 8:**
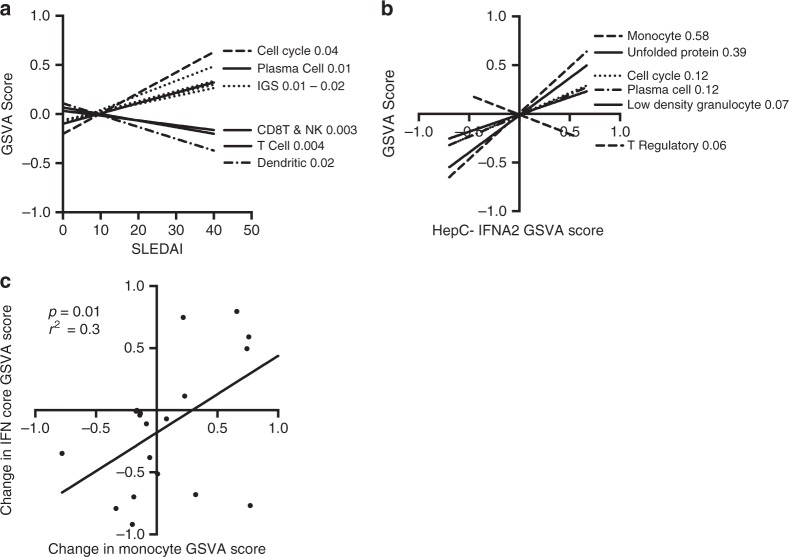
Linear regression analysis demonstrates the IFN signature is most closely related to monocyte cell surface transcripts. Linear regression analysis using SLEDAI values from the patients of 5 SLE WB and 2 SLE PBMC datasets and the patient GSVA enrichment scores for cell type specific signatures (Supplemental Table 12). **a** Cell types or signatures with significant non-zero slopes (*p* < .05) related to SLEDAI by linear regression analysis in at least half of the datasets which had determinable GSVA scores (Supplementary Data 13) were used to determine overall significance of the regression lines and the r^2^ predictive values for all 7 SLE datasets with available SLEDAI information (Supplementary Data 14, 15). Cell type or process GSVA enrichment categories with linear regression *p* values < .05 are shown and *r*^2^ predictive values are listed after the cell type or process. **b** Representative plot using the HepC-IFNA2 signature for the linear regression analysis between the IFN signature with overlapping transcripts to the cell type or process signatures removed (Supplementary Data 16) and the cell type or process GSVA enrichment score for the patients from 10 SLE WB and PBMC datasets (Supplementary Data 17, 18). Cell types or signatures significantly (*p* < .05) related to HepC-IFNA2 score in at least half of the datasets which had determinable GSVA scores were used to determine overall regression lines for all 10 datasets. *r*^2^ predictive values are listed after the GSVA enrichment category. Supplementary Data 19–28 show relationships and linear regression analysis for the other IFN signatures. **c** For time-course dataset GSE72747, linear regression analysis was done for the change in the core IFN GSVA scores versus the change in monocyte cell surface scores between 0 and 12 weeks and 12 and 24 weeks

Linear regression analysis was used to determine the relationship between the IGS and cell types, cellular processes, clinical measures and SLEDAI. As anticipated, IFN core (*r*^2^: 0.07–0.14), and IFNB1 (*r*^2^: 0.01–0.29) exhibited a positive relationship (*p* < .05) with SLEDAI, but the *r*^2^ predictive values were low. For individual datasets, the most consistent positive relationship with SLEDAI (a non-zero slope; *p* < .05) was with genes involved in regulation of the cell cycle (*r*^2^ range: 0.02–0.18), plasma cells (*r*^2^: 0.01–0.17) and anti-double stranded DNA antibodies (*r*^2^: 0.06–0.21). T cell, CD8 T-Natural Killer (NK) cell, and dendritic cell GSVA enrichment scores had significant negative relationships with SLEDAI (*p* < .05) in most datasets but also with low ranges of predictive *r*^2^ values; T cell (0.09–.321), CD8-NK (.06–.26), and dendritic cell (.02–.2) (Fig. 8a**;** Supplementary Data 14, 15**)**.

To determine whether the IGS detected in SLE patients was related to a specific type of hematopoietic cell or process, linear regression analysis was carried out between the GSVA enrichment scores for cell signatures and processes and the IGS in each SLE patient from ten SLE WB and PBMC datasets. The strongest relationship to the IGS was to monocyte surface transcripts with a significant non-zero slope (*p* < .0001) and a range of *r*^2^ values of 0.29–0.58. Other categories with a significant relationship (*p* < .05) to most IGS, but with a lower range of predictive values were the cell cycle (.12–.28), plasma cells (.12–.23), the unfolded protein response (.15–.39) and low density granulocytes (.03–.07) (Fig. 8b; Table 1**;** Supplementary Fig. 8**;** Supplementary Data 13; 17–28). Inconsistent associations between T cell and dendritic cell categories and the IGS were observed. The association of the monocyte signature with the IGS suggested that monocytes and the IGS would change synchronously. In order to test this, the change in monocytes versus the change in the IFN core signature for time-course dataset GSE72747 was carried out. Figure 8c demonstrated a significant relationship (*r*^2^ = .3; *p* < .05) by linear regression analysis between the change in monocytes and the change in IFN core signature. Insufficient plasmacytoid dendritic cell specific transcripts made GSVA unreliable, but *CLEC4C* (BDCA-2) and *NRP1* (BDCA-4) transcripts were decreased in 6/10 and 2/10 SLE WB and PBMC datasets respectively, and not uniformly associated with the IGS (Supplementary Data 29).

**Table 1:** Linear regression *r*^2^ values for IFNs and cell type signatures

	Cell cycle	Plasma cells	Unfolded protein response	Mono cell surface	Low density granulocyte	B cells	T Reg	T cells	CD8 T and NK
MS-IFNB1	0.28	0.23	0.15	0.29					
HepC-IFNA2	0.12	0.12	0.39	0.58	0.07		*0.05*	0.01	0.02
IFNA2	0.27	0.23		0.34	0.04				
IFNB1	0.19	0.19	0.28	0.35	0.03	0.06			
IFNW1	0.24	0.04	0.31	0.40	0.05			0.01	
IFN Core	0.27	0.23	0.24	0.34	0.04			0.01	

The strong relationship of the IGS to the monocyte signature could be related to a stronger relative IGS expression in monocytes compared to B and T cells. In order to test this, differential expression (DE) analysis was performed between CD4 T cells, CD19 B cells, and CD14 monocytes from active (SLEDAI ≥ 6) SLE patients obtained from publicly available microarray datasets (Supplementary Data 1). Comparison of expression of the IFN core transcripts revealed that monocytes overexpressed (fold change > 2, false discovery rate < .05) three times as many IGS transcripts as T cells (92 to 28 transcripts) or B cells (94 to 29 transcripts). Transcripts increased by more than 16 fold change in SLE monocytes compared to both T and B cells included *IL1RN*, *SERPING1*, *PLSCR1*, *EIF2AK2*, *JAK2*, and *CXCL10*. *LAMP3* was overexpressed in SLE T cells compared to SLE monocytes and B cells and *APOBEC3B*, *STAP1*, and *SPIB* were overexpressed in SLE B cells compared to SLE monocytes and T cells (Supplementary Data 30). The IGS transcripts not DE between purified cells were also of interest; *IFI27*, *IFI44L*, *IFIH1*, *IFIT3*, *OASL*, *RSAD2*, *SPATS2L*, and *USP18* were all highly DE when each cell type was compared to controls, but not DE between SLE T cells, B cells or monocytes (Supplementary Data 30 and 31). Comparison of these eight genes to the individual signatures used in Fig. 8 showed that the HepC-IFNA2 signature had none of these transcripts and the MS-IFNB1 signature had all eight which may explain in part the weaker predictive relationship (*r*^2^ = .29) between the MS-IFNB1 signature and monocytes.

**Fig. 9:**
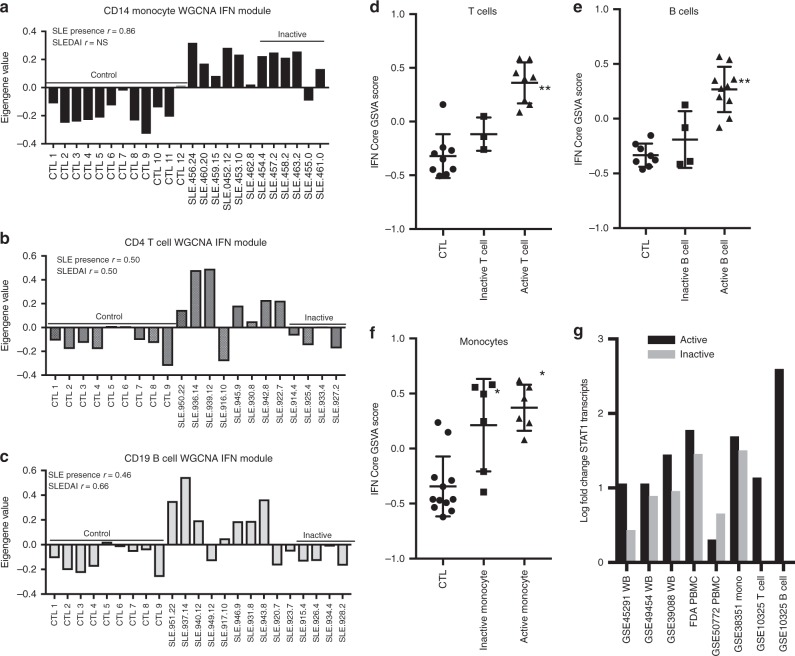
Monocytes from inactive SLE patients have an interferon signature and elevated STAT1 transcripts. **a**–**c** Weighted Gene Coexpression Network Analysis (WGCNA) was performed on datasets GSE38351 CD14+ monocytes (6 active (SLEDAI ≥ 6), 6 inactive (SLEDAI < 6), 12 control), GSE10325 CD4+ T cells (8 active, 4 inactive, 9 control) and GSE10325 CD19+ B cells (10 active, 4 inactive, 9 control) (Supplementary Data 1) and individual patient eigengene values are shown for the IFN module from each dataset. The modules were correlated to presence of SLE disease (versus control) or the SLEDAI and Pearson r values are shown for significant correlations for each WGCNA dataset (*p* < .05). NS means not significant (Supplementary Data 32 for WGCNA IFN modules). SLEDAI values for each patient are listed at the end of the patient number with controls and patients with inactive disease noted by underlined text. GSVA enrichment scores were calculated using the IFN core signature for SLE and control samples of (**d**) CD4+ T cells, (**e**) CD19+ B cells, and (**f**) CD14+ monocytes. Tukey’s multiple comparison test was used to determine significant differences between mean GSVA scores between controls, inactive and active patients. Asterisks (*) indicates a *p*-value of <.05 between active SLE and control or inactive SLE and control; double asterisks (**) indicates a *p*-value of <.05 between active SLE and inactive SLE and active SLE and control. **g** Datasets of SLE WB, PBMC, purified CD14+ monocytes, T and B cells were divided into active (SLEDAI ≥ 6) and inactive (SLEDAI < 6) for DE analysis to controls. The log fold change for *STAT1* is reported for each active and inactive dataset

To explore the relationship between monocytes and the IGS in greater detail, WGCNA analysis was carried out on purified CD14 monocytes, CD4 T cells and CD19 B cells from SLE patients with active and inactive disease. A discrete IGS module was delineated for monocytes and T cells and the IGS in CD19 B cells grouped with cell cycle transcripts (Fig. 9a–c; Supplementary Data 32). All three modules showed significant Pearson correlation (*p* < .05; *r* ≥ .5) to the presence of SLE (versus control) but only the IGS modules from T and B cells also showed significant correlation to SLEDAI (*p* < .05; *r* ≥ .5). Eigengene values for monocytes from SLE patients with inactive disease had mostly positive values for the IFN module in contrast to the negative values for T and B cells from SLE patients with inactive disease. GSVA enrichment scores using the IFN core signature showed that T cells (Fig. 9d) and B cells (Fig. 9e) from inactive SLE patients displayed low or absent IGS, whereas monocytes from inactive SLE patients had IGS similar to monocytes from active SLE patients (Fig. 9f). As IFNs have been shown to induce the transcription of *STAT1* in monocytes^48^ and increased transcription could lead to an increase in unphosphorylated STAT1 and a prolonged IGS in the absence of IFNs^49^ transcripts for *STAT1* were evaluated and shown to be elevated in both active and inactive SLE WB, PBMC, and monocyte datasets, but not in T and B cells from inactive SLE patients (Fig. 9g). Thus, monocytes in WB and PBMC may retain the IGS in SLE patients with low disease activity, and also relatively over-express more IGS transcripts than T or B cells.

## Discussion

This study presents a novel approach to interrogate the IGS in SLE microarray datasets using reference datasets. The use of microarray data from unrelated yet relevant datasets as a tool for microarray dataset interrogation is an important advance, since it does not rely on prior characterization or knowledge of any genes, and also focuses the analysis on gene changes that have been shown to be operative in human samples. Using this approach, we demonstrated strong enrichment for IFNB1 in SLE skin and synovium and importantly showed a strong similarity between signatures in patients treated chronically with IFNB1 and the SLE WB signature. Moreover, we were able to relate the IGS to monocytes in the analyzed samples.

Z score calculations and GSVA enrichment scores demonstrated the likely role of IFNB1 in SLE pathogenesis and suggested that targeting this IFN in lupus skin and synovium may be more beneficial than blocking IFN in SLE patients with LN. The finding that the kidneys differ from skin and synovium is unexpected and could not be anticipated from the blood analysis demonstrating the important contributions of tissue samples to this and future work. Single cell analysis of hematopoietic cells derived from the kidneys of LN patients demonstrated a low IGS in cells from most patients and this together with our data may suggest that the IFN signaling pathway may not be as prominent in this tissue compared to skin and synovium^50^. It is interesting to note that both skin and synovium are rich in fibroblasts, an important IFNB1 producing cell type^1^ and that constitutive IFNB1 production may provide a background of IFN in these tissues, whereas the normal kidney has relatively few fibroblasts.

The greater association between the MS-IFNB1 signature and the SLE IGS signature is of particular note. The much higher Z scores calculated using the MS-IFNB1 signature for all WB, PBMC, and SLE affected tissues in comparison to the calculated GSVA enrichment scores is related to the increased overlap of decreased transcripts between the MS-IFNB1 signature and the signature in SLE patients. Long-term exposure to IFNB1 in MS patients led to a decrease in transcripts such as *CD1C*, *CD160*, *IGF1R*, and *TNFRSF9* that were also seen in SLE patients. All of these molecules participate in cellular activation and inhibition of them after long-term exposure to IFNB1 could suggest a shared downregulatory mechanism between MS patients treated with IFNB1 and SLE patients.

Although this work showed strong enrichment of IFNB1 in SLE, it does not preclude a role for the IFNAs. Indeed, IFNB1 itself has been shown to induce the expression of IFNAs^51,52^. The two-step model of type I IFN induction by viruses, TLR or other cytosolic pattern recognition receptors establishes that the activation of the constitutively expressed IRF3 in the cytoplasm leads to the initial induction of only *IFNB1*. The IFNB1 protein acts on the IFNA/B receptor to induce IRF7 expression by activating ISGF3 in the cytoplasm leading to the induction of *IFNA*s^53–55^. Although little has been published regarding *IFNW1* in humans, it is among the most induced genes, along with *IFNA2* and *IFNB1*, after plasmacytoid dendritic cell treatment with TLR7 agonists^56^.

The IFNG signature had significant effect size and Z scores for all SLE tissues and most peripheral datasets albeit lower than the type I signatures. The induction of type I IFNs in response to virus initiates a cascade of events leading to the recruitment/activation of CD8 T cells and NK cells^57^. While *IFNG* is induced in CD8 T cells, NK cells constitutively express *IFNG* transcripts^58^ and NK cells are not easily discernible from CD8 T cells by microarray expression. In lupus mouse models, IFNG appears to play a more prominent role than in humans, and we hypothesize the presence of IFNG may represent a late stage response to the inappropriate induction of type I IFNs to sterile inflammatory stimuli.

This study demonstrates clearly that inactive SLE patients have a readily detectable IGS and that some SLE patients over time may change their IGS status. We were able to demonstrate the gain or loss of the IGS in approximately 30% of 119 subjects in contrast to previous results showing little change in 11 SLE patients over time^15^. This change in status in the absence of intense immunotherapy suggests that the IGS is not stable during the disease process in one third of SLE patients. Petri et al.^15^ had previously shown in 66 SLE patients that increased SLEDAI was associated with an increase in a 3 gene IGS, but our data from more than 2000 patients suggests there is not a relationship between SLEDAI and the IGS. Similar to data from this group, our data also showed no predictable relationship between the SLEDAI and IGS. In ten SLE LN patients the IGS did not change synchronously with the SLEDAI and we further demonstrated that the change in IGS was associated with a change in monocytes.

Because of the high degree of heterogeneity in both SLE patients^59^ and in microarray dataset platforms, processing and controls, we chose a meta-analysis approach in order to understand and interpret the relationship between gene expression signatures to each other and disease activity. Linear regression analysis of the SLEDAI and GSVA scores for cell types, cellular processes, or IGS for seven SLE datasets showed the strongest relationship to the SLEDAI was expression of genes regulating the cell cycle and plasma cells. This was reassuring as this cell cycle signature was taken from a WGCNA plasma cell module in SLE CD19 B cells correlated to SLEDAI^60^ and plasma cells have been shown to correlate with SLEDAI^61–63^. The IFN core, IFNW1, and IFNB1 signatures had low positive correlations with SLEDAI, and as was the case for the cell cycle and plasma cell signatures, had low predictive value for the SLEDAI.

A predictive relationship across ten SLE WB and PBMC datasets (2037 patients) was determined for all the IGS and monocyte cell surface transcripts with a range of *r*^2^ predictive values of .29–.58. This suggested that the IGS is most related to the increased presence of monocytes expressing the IGS. Three times as many transcripts from the IFN core signature were enriched in monocytes relative to T cells and B cells. Although we found that some members of the IGS in SLE were highly overexpressed in SLE monocytes, some of the most overexpressed transcripts when SLE patients were compared to controls were not over-expressed in SLE monocytes compared with SLE T and B cells. Support for monocytes having a greater intensity IGS was recently claimed in experiments in which the log signal ratios of a 20 gene IGS was compared between purified T cells, B cells, and monocytes in SLE patients^64^.

In addition to monocytes from active SLE patients expressing a greater intensity for 2/3 of the IFN core transcripts, T and B cell WGCNA-derived IFN modules correlated significantly to SLEDAI whereas the CD14 monocyte IFN module did not. The presence of an IGS in CD14 monocytes from inactive patients, but not in CD4 T and CD19 B cells from inactive patients supports the idea that monocytes are maintaining the IGS in inactive SLE patients. One explanation for this may be the increased *STAT1* transcripts found in inactive SLE WB, PBMC and monocyte datasets but not the in inactive SLE CD4 T or CD19 B cells. A prolonged IGS in monocytes in the absence IFN could also explain why some patients with IGS signatures had no IFNA detected using an ultrasensitive ELISA^65^. Another possible explanation for how monocytes may maintain an enhanced IGS derives from experiments treating human monocytes with a combination of TNF and IFN on a background of TLR signaling. IFN treatment in this context led to epigenetic changes allowing for a much greater IGS than when cells were stimulated with IFN alone^66^. Thus, the presence of inflammatory cytokines such as TNF, along with nucleic acid-containing immune complexes capable of signaling through TLRs may account for the prolonged IGS seen in monocytes even when disease activity is low. It is also notable that variety of DE genes can be detected in myeloid cells of subjects with inactive SLE, whereas T cells or B cells of inactive SLE patients showed no DE transcripts compared to controls  at a false discovery rate of 0.2^67^.

IFNB1 presents an intriguing target for SLE therapy because of the predominance of its signature in SLE affected tissues, its unique signaling properties and cellular expression, and its potential role in B cell development and tolerance^68,69^. However, this work demonstrated that the IGS will likely not correlate with the SLEDAI disease measurement and that a prolonged IGS in monocytes could make interpretation of the IGS as a measure of disease activity or the immediate presence of IFN difficult. The potential benefit of targeting IFNB1 must be considered within the practical limitations of disease measurement indices used in SLE clinical trials.

## Methods

### Compilation of gene expression data from SLE patients

Data were derived from publicly available datasets and collaborators. See Supplementary Data 1 for individual accession codes.

### Differential gene expression (DE)

DE was done for each dataset of SLE patients and controls. GCRMA normalized expression values were variance corrected using local empirical Bayesian shrinkage before calculation of DE using the ebayes function in the open source BioConductor LIMMA package^70^ (https://www.bioconductor.org/packages/release/bioc/html/limma.html). Resulting *p*-values were adjusted for multiple hypothesis testing and filtered to retain DE probes with an False Discovery Rate < 0.2^71^. This cutoff was employed a priori to increase the number of genes that would subsequently be analyzed, with the understanding that even though the number of false positives might be increased, fewer false negatives would be excluded from the analysis. The heterogeneity in SLE patient blood samples has been previously demonstrated^59^ and as a practical matter, signatures for low density granulocytes and plasma cells are sometimes not detectable in limma analysis of populations depending on the specific patient make-up. A False Discovery Rate of 0.2 allows detection of cell types and processes, which may not be found in all SLE patients, but that contribute significantly to the disease state in subpopulations of patients.

### Gene set variation analysis (GSVA)

GSVA^40^ (V1.25.0) software package is an open source package available from R/Bioconductor and was used as a non-parametric, unsupervised method for estimating the variation of pre-defined gene sets in patient and control samples of microarray expression datasets (www.bioconductor.org/packages/release/bioc/html/GSVA.html). The inputs for the GSVA algorithm were a gene expression matrix of log2 microarray expression values and pre-defined gene sets co-expressed in SLE datasets. Enrichment scores (GSVA scores) were calculated non-parametrically using a Kolmogorov Smirnoff -like random walk statistic and a negative value for a particular sample and gene set, meaning that the gene set has a lower expression than the same gene set with a positive value. The enrichment scores (ES) were the largest positive and negative random walk deviations from zero, respectively, for a particular sample and gene set. The positive and negative ES for a particular gene set depend on the expression levels of the genes that form the pre-defined gene set.

Random Group (Gr) 1 and Random Group (Gr) 2 signatures were determined by first assigning random numbers to the list of DE genes (False Discovery Rate < 0.2) from dataset GSE49454 in Microsoft® Excel® for Mac 2011 using the formula = *rand()*, and then sorting on ascending genes and taking the first 100 genes. This was performed twice to generate Random Gr1 and Random Gr2 signatures. Gene symbols for these signatures are listed in Supplementary Data 2. Enrichment modules containing cell type and process specific genes (Supplementary Data 12) were created through an iterative process of identifying DE transcripts pertaining to a restricted profile of hematopoietic cells in a majority of the 13 SLE microarray WB and PBMC datasets in Supplementary Data 1 and checked for expression in purified T cells, B cells, and Monocytes to remove transcripts indicative of multiple cell types; transcripts were researched by literature mining. In the case of the cell cycle, unfolded protein response and plasma cell modules, genes were initially identified through the DE analysis and WGCNA created modules correlated to SLEDAI from CD19 and CD20 B cells as reported in Grammer et al.^60^; these genes were identified through literature mining and STRING interactome analysis as belonging to these categories and their DE was confirmed in the 13 SLE WB and PBMC datasets used in these studies. In order to have a significant overlap, a minimum number of three transcripts for each category had to be found in each dataset and (Supplementary Data 13) was used based on calculating a 20% error rate for one transcript, 4% error rate for two transcripts and an error rate of .8% for three transcripts. GSVA enrichment modules used for linear regression analyses had overlapping transcripts between the IFN signatures and the cell type specific signatures removed and are shown in Supplementary Data 16.

Differences between healthy controls and SLE patient GSVA enrichment scores were determined using the Welch’s *t*-test for unequal variances in PRISM 7.0 v7.0c. In order to quantitate the difference between the SLE and healthy control groups, the Hedge’s *g* effect size was determined using the Effect Size Calculator for *T*-Test at the website Social Science Statistics, www.socscistatistics.com/effectsize/Default3.aspx.

### Z score analysis

Z score calculations were employed to identify and compare the enrichment of specific signatures in SLE and control datasets. For each regulator, an activation z-score was calculated strictly from the experimentally observed information provided for the downstream targets. Reference datasets were used to determine the identity and direction (increased or decreased) of downstream targets. The formula **Z** **=** **x (σ**_**x**_**)**^**−1**^ **=** **Σ**_**i**_**w**_**i**_**x**_**i**_ (**√Σ**_**i**_**w**_**i**_^2^**)**^**−1**^ was used to calculate Z scores with edge weights set to 1. Z scores above or below 1.96 are significant at the 95% confidence level and Z scores above or below 2.54 are significant at the 99% confidence level. SLE WB and PBMC datasets were divided into patients with SLEDAI ≥ 6 (active) and patients with SLEDAI < 6 (inactive).

### Reference and control datasets

The first reference dataset used was the transcripts (False Discovery Rate < .01, Fold Change > 2) from the in vitro treatment of healthy, human PBMC with 0.6 pM IFNA2b, IFNB1a, IFNW1, IFNG, IL12 or TNF differentially expressed compared to control-treated PBMC. To eliminate differences in genetic background, a single donor was used for these experiments (Table S2, Waddell et al.^39^). The second reference dataset used was the IFNB1 (MS-IFNB1) signature-induced in vivo in the whole blood of 58 Multiple Sclerosis patients treated with IFNB1 (Avonex, Betaseron or Rebif) for one to two years compared to 62 MS Patients not treated with IFNB1 from Table S1^41^. The third reference dataset used was the IFNA signature-induced in 4 HepC patients treated with recombinant IFNA for 6 h compared to their PBMC before the injection of recombinant IFNA from Table 2^42^ for the HepC-IFNA2 signature. Published transcripts of PBMC from patients with sepsis DE to controls (Table S2)^43^ and skin biopsies from patients with dermatomyositis DE to controls (Table S2)^44^ were used as comparators for Z score calculations. The reference dataset for the alternative IFNB1 signaling pathway was taken from the IFNB1-induced signature through the IFN-Alpha/Beta Receptor 1 in IFN-Alpha/Beta Receptor 2 deficient peritoneal exudate cells (Supplementary Data 2)^30^. Genes were translated to human gene symbols and the increased transcripts were used to determine GSVA scores.

### Weighted gene coexpression network analysis

Weighted Gene Coexpression Network Analysis (WGCNA)^72^ is an open source package for R available at https://horvath.genetics.ucla.edu/html/CoexpressionNetwork/Rpackages/WGCNA/.

Log2 normalized microarray expression values for WB, PBMC, purified T cell, B cell, or monocyte datasets were filtered using an IQR to remove saturated probes with low variability between samples and used as inputs to WGCNA (V1.51). Adjacency co-expression matrices for all probes in a given set were calculated by Pearson’s correlation using signed network type specific formulae. Blockwise network construction was performed using soft threshold power values that were manually selected and specific to each dataset in order to preserve maximal scale free topology of the networks. Resultant dendrograms of correlation networks were trimmed to isolate individual modular groups of probes, labeled using semi-random color assignments, based on a detection cut height of 1, with a merging cut height of 0.2, with the additional use of a partitioning around medoids function. Final membership of probes representing the same gene into modules was based on selection of greatest scale within module correlation against module eigengene values. Correlation to the presence of SLE disease (versus control) or the disease measure SLEDAI were performed using Pearson’s r against module eigengenes, defining modules as either positively or negatively correlated with those traits as a whole.

### DE gene expression in SLE patients with multiple time-points

One-way analysis of variance (ANOVA) was used to compare means of two or more samples (using the F distribution). The statistic fit2$F and the corresponding fit$F. *p* value were used to combine the pair-wise comparisons into one F-test. This is equivalent to a one-way ANOVA for each gene except that the residual mean squares have been moderated between genes. For the GSE88885 dataset, a subset of patients on standard of care therapy and placebo from the Illuminate 1 clinical trial had time-course microarray expression data; 86 placebo treated SLE patients at *t* = 0, *t* = 16 and *t* = 52 weeks and 16 healthy controls were analyzed together. For GSE88886, a subset of placebo patients on standard of care therapy from the Illuminate 2 clinical trial with time-course microarray data, 33 placebo treated SLE patients with time points at *t* = 0, *t* = 16 and *t* = 52 weeks and 12 healthy controls were analyzed together. For GSE72747 all ten patient values at *t* = 0, *t* = 12 and *t* = 24 weeks and 46 healthy controls from GSE39088 were analyzed together. Significant changes in IGS were determined to be a standard deviation (SD) of 0.2 by calculating the SD of the healthy controls for each signature and using the highest SD as a measure of significance.

### Other Statistical analyses

GraphPad PRISM 7 version 7.0c was used to perform linear regression analysis, calculation of *r*^2^ values and Tukey’s multiple comparison analysis for ANOVA. Average and SD were calculated using Microsoft® Excel® for Mac 2011. The built-in ANOVA function in R was used to compute two-way ANOVA *p*-values.

### Reporting Summary

Further information on experimental design is available in the Nature Research Reporting Summary linked to this article.

## Supplementary information

Supplementary Information

Description of additional supplementary items

Reporting Summary

Supplementary Data 1–32

## Data Availability

All microarray datasets in this publication are available on the NCBI’s database Gene Expression Omnibus (GEO) (https://www.ncbi.nlm.nih.gov/geo/) except the FDA PBMC dataset. See Supplementary Data 1 for individual accession codes. The FDA PBMC microarray dataset is available for download in the Supplementary Materials, File S1 of Labonte et al.^67^

## Code availability

All software used in this publication is open source, freely available for R. Additionally, example code used in this paper for LIMMA, GSVA and WGCNA are available at figshare^73–75^. Files can be accessed at https://figshare.com/articles/AMPEL_ BioSolutions_LIMMA_DIfferential_Expression_Analysis_Code_rtf/7780247, https://figshare.com/articles/AMPEL_BioSolutions_Gene_Set_Variation_Analysis_Code/7780457 and https://figshare.com/articles/AMPEL_BioSolutions_Weighted_ Correlation_Network_Analysis_WGCNA_Code_txt/7778552.
