# Supplementary material for: Gene expression analysis delineates the potential roles of multiple interferons in systemic lupus erythematosus
Source: Commun Biol. 2019 Apr 23;2:140. doi: 10.1038/s42003-019-0382-x (PMC6478921; doi:10.1038/s42003-019-0382-x)
Supplement: Supplementary file 1 — Supplementary Information [file 42003_2019_382_MOESM1_ESM.pdf]

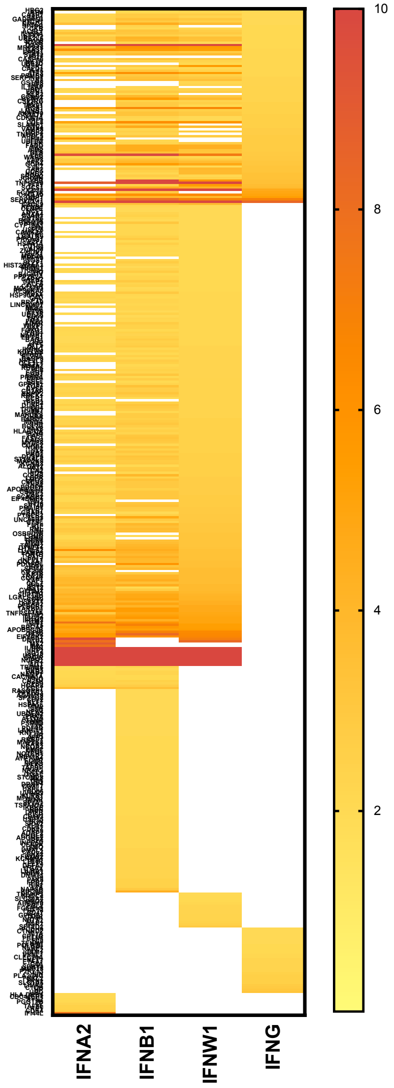

Supplementary Figure 1. Waddell et al.,[1] transcripts from the in vitro treatment of PBMC with IFNA2, IFNB1, IFNW1 and IFNG. Transcripts increased by a minimum fold change of 2 at a false discovery rate of 0.05 compared to mock treated PBMC are shown. Unique transcripts for IFNA2, IFNB1, IFNW1 and IFNG were determined by comparison of the four signatures. The heatmap scale represents fold change. Gene symbols for each signature are listed in **Supplementary Data 2**.

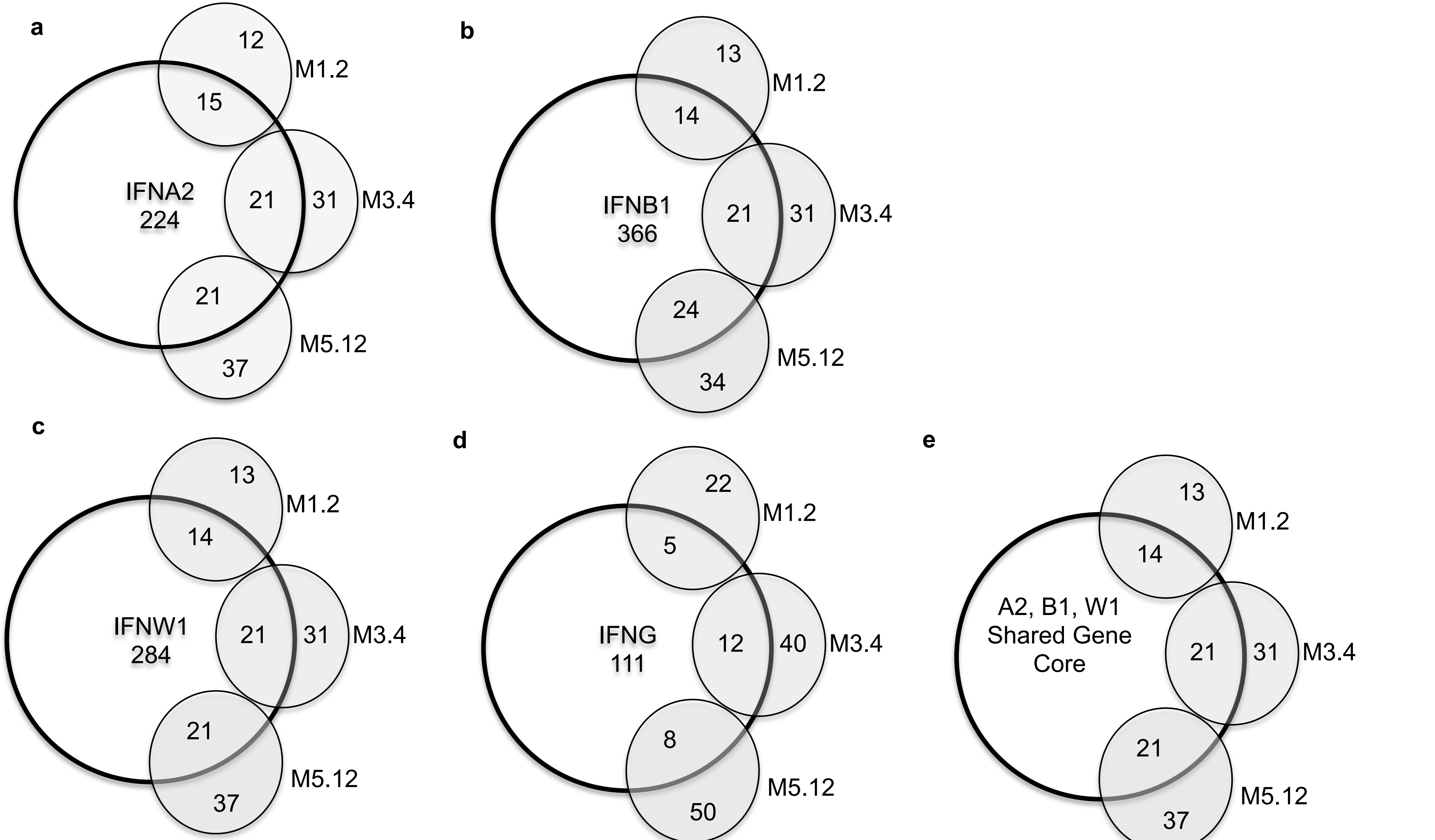

Supplementary Figure 2. Chiche-Chaussable Modules Do Not Reflect a Specific Sub-type of IFN. Overlap of the three Chiche-Chaussabel interferon modules (IFN-M)[2] with the Waddell transcripts[1] induced by (**a**) IFNA2, **(b)** IFNB1, (**c**) IFNW1, and (**d**) IFNG. Each IFN-M overlapped the IFNA2, IFNB and IFNW1 signatures with the same genes except IFI44L from M1.2 was only in IFNA2 and DRAP1, NBN and IRF9 from M5.12 were only found in the IFNB1 induced transcripts. (**e**) Overlapping genes were found within the core IFN genes, not the unique IFN signatures.

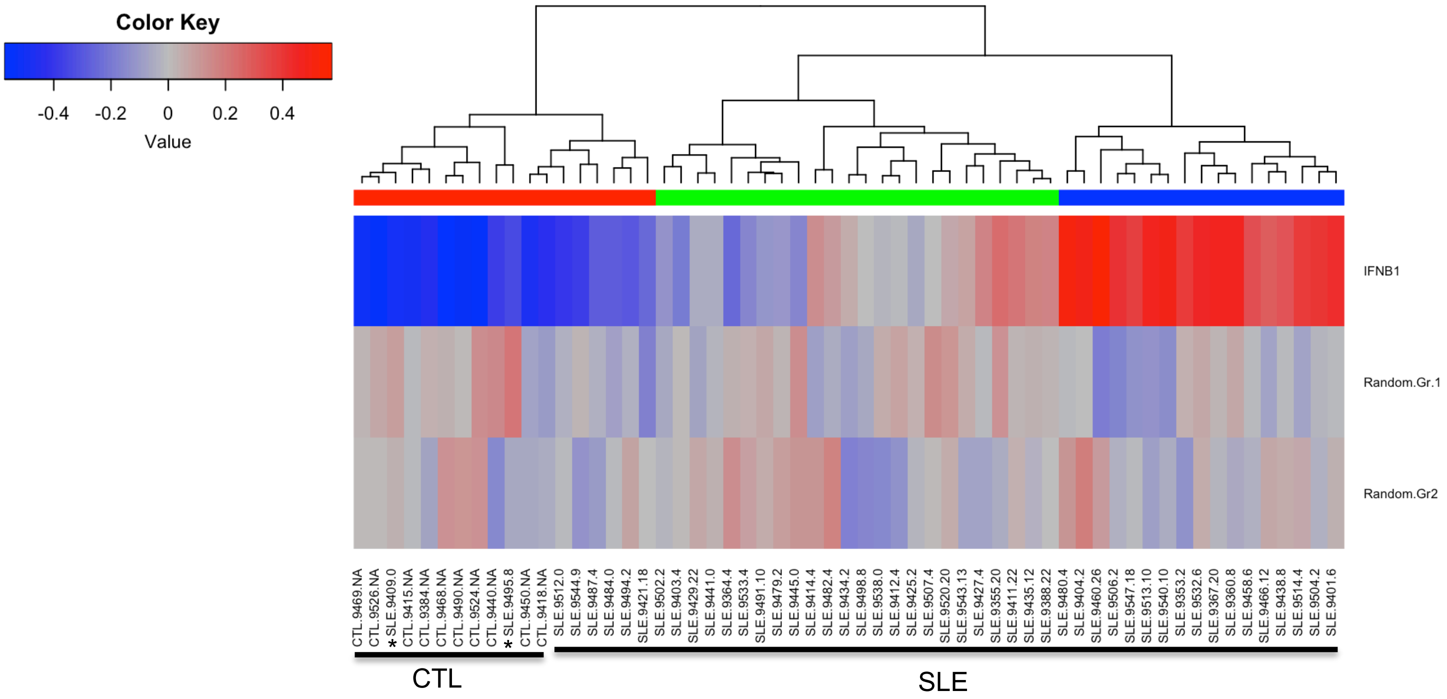

Supplementary Figure 3. GSVA Enrichment Using Random Genes Does Not Separate SLE Patients from Controls. Heatmap visualization of the GSVA enrichment scores for the Waddell IFNB1 increased transcripts (IFNB1) and two groups of random, not co-expressed transcripts derived from random sorting of dataset GSE49454 DE transcripts (**Supplementary Data 2 and Methods**). Enrichment scores were calculated using these groups for all patients and controls in dataset GSE49454 (n = 46).

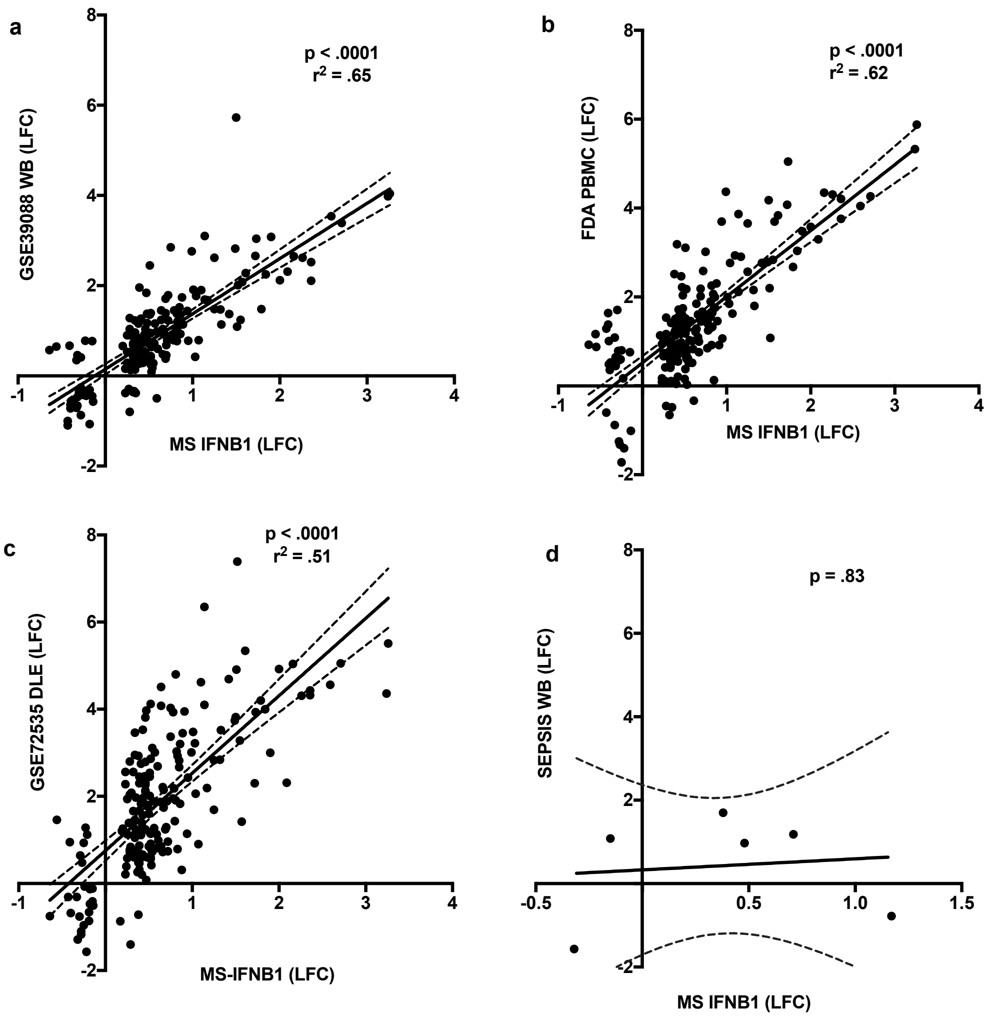

Supplementary Figure 4. MS-IFNB1 Signature in Multiple Sclerosis Patient Whole Blood Confirmed Strong IFNB1 Signature. Linear Regression Analysis using the MS-IFNB1 Signature of increased and decreased transcripts with (**a**) SLE Active (SLEDAI ≥ 6) WB, (**b**) SLE Active PBMC, (**c**) DLE, and (**d**) Sepsis.

a WB GSE49454

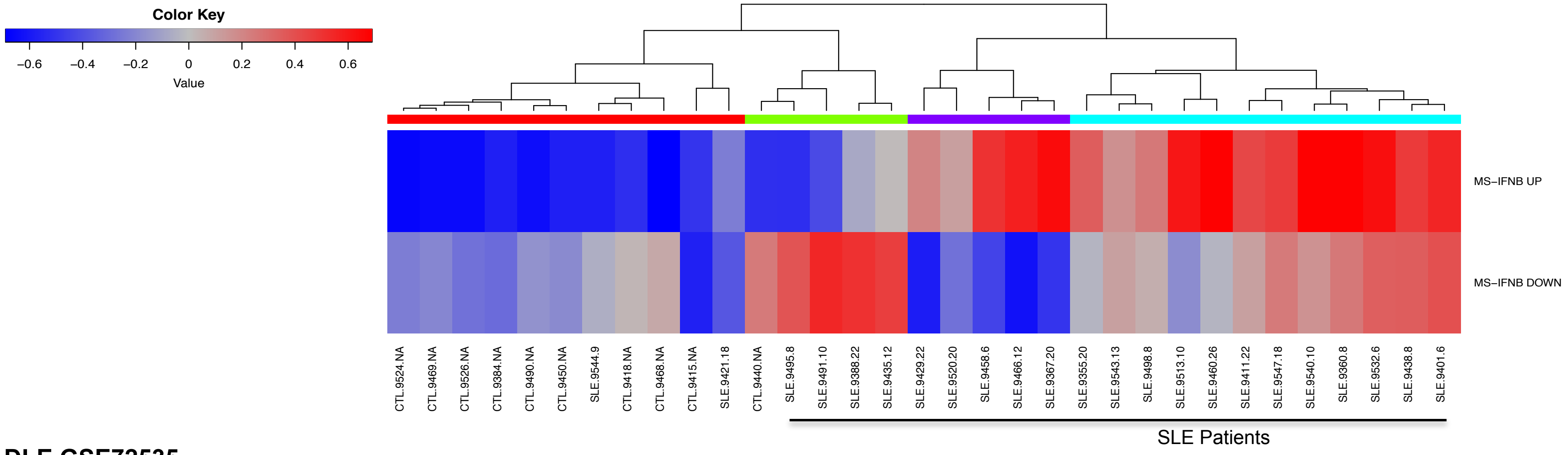

b DLE GSE72535

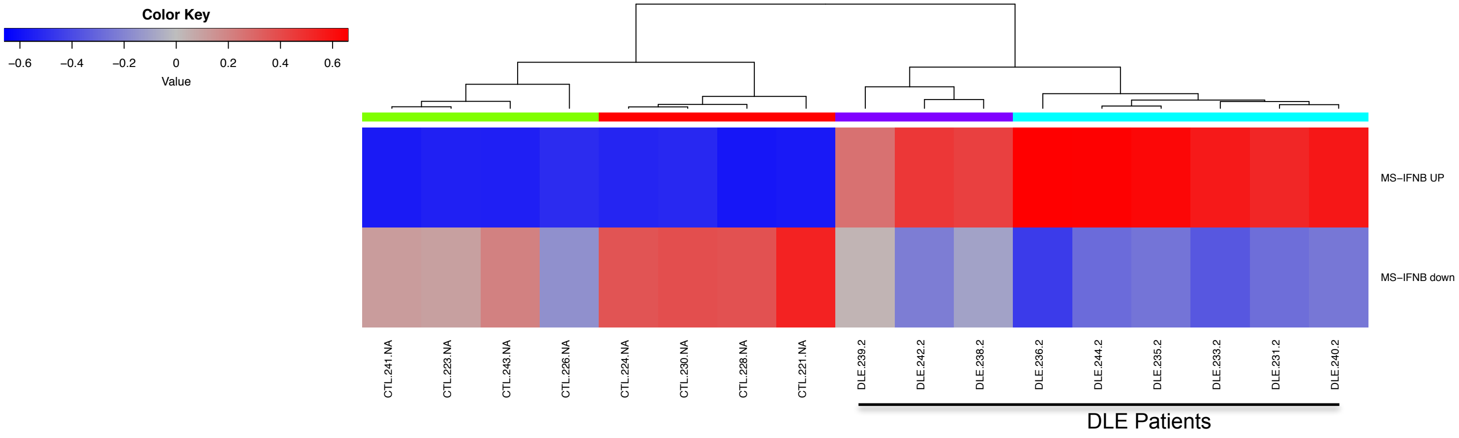

Supplementary Figure 5. MS-IFNB1 Signature Separates SLE Cells and Tissues. GSVA using the MS-IFNB1 Signature. Increased (IFNB UP) and decreased (IFNB DOWN) transcripts (DE to untreated multiple sclerosis patients) separated SLE patients from controls in (**a**) WB GSE49454 active (SLEDAI ≥ 6) SLE patients (n = 23) and (**b**) DLE GSE72535 (n = 9).

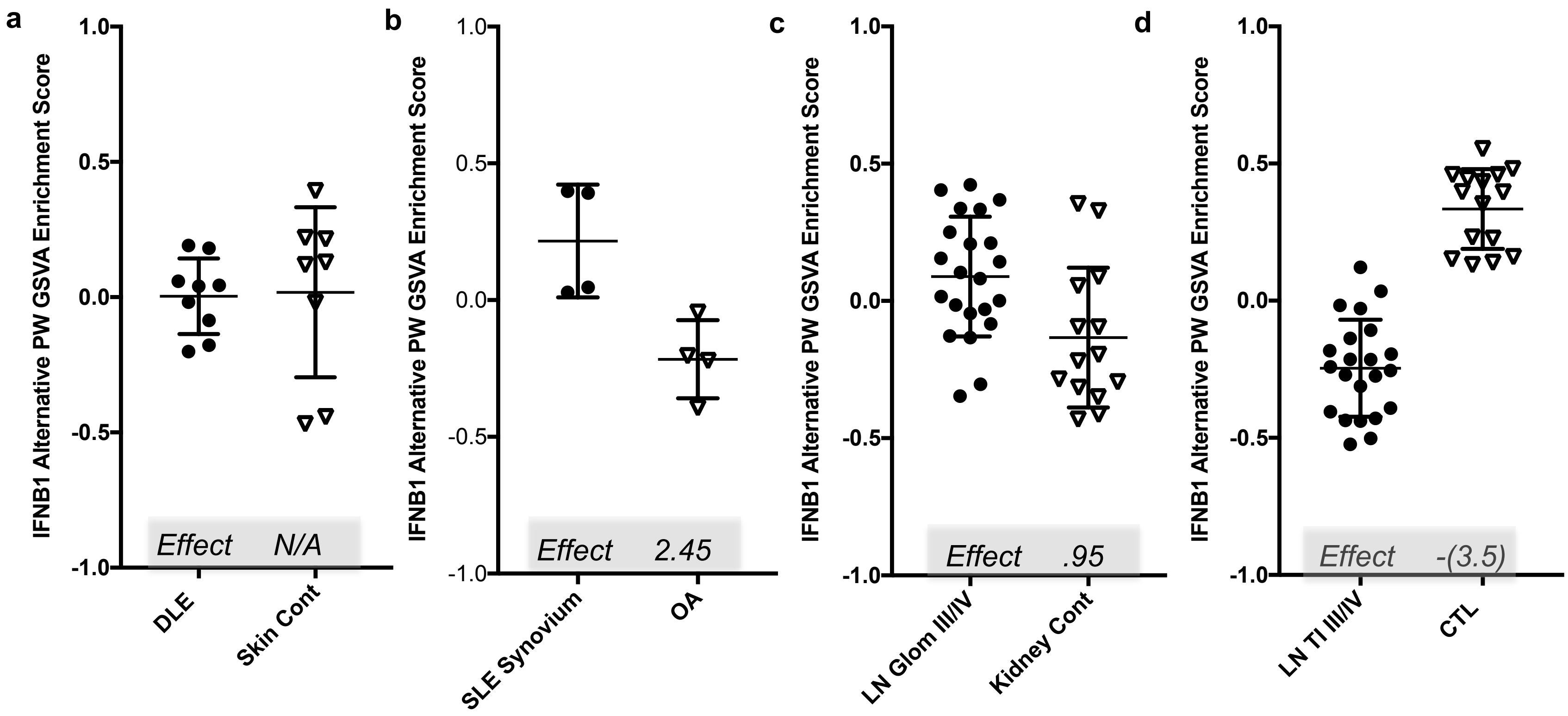

Supplementary Figure 6. The Alternative IFNB1 Downstream Signaling Pathway Does Not Predominate in SLE Tissues. Murine IFN alpha/beta receptor 2 deficient mice were injected with IFNB1 into the peritoneum and peritoneal exudate cells (PEC) were isolated for microarray expression analysis to control PEC[3]. Increased transcripts induced by IFNB1 signaling through the IFN alpha/beta receptor 1 only were used as a GSVA enrichment group to determine if the alternative pathway of IFNB1 signaling was contributing to gene regulation in **(a)** DLE, **(b)** SLE synovium, **(c)** LN Glom class III/IV, and **(d)** LN TI class III/IV. Hedge's G effect size measures (Effect) are shown for tissues with significant ($p < .05$) differences between the mean GSVA enrichment scores between SLE affected and control tissues by Welch's t-test. N/A = not applicable due to insignificant Welch's t-test value.

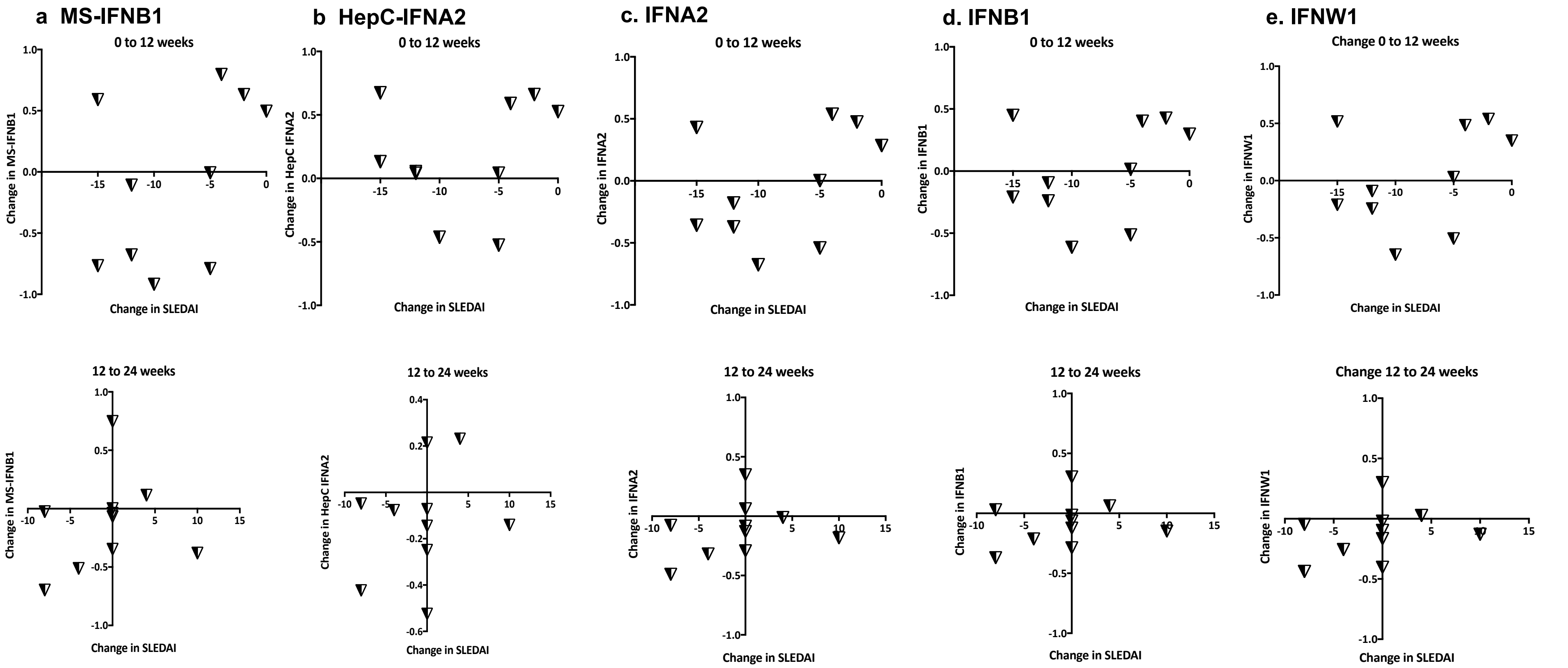

Supplementary Figure 7. The IGS and SLEDAI Do Not Change Synchronously Ten SLE lupus nephritis patients with SLEDAI > 6 (GSE72747) had Ftest DE analysis using time 0, 12 and 24 week time points Treatment with high dose immunosuppressive was begun after time zero and continued for 12 weeks; at 12 weeks all patients were switched to lower dose / maintenance therapy; healthy controls from the GSE39088 dataset were included in the analysis. Graphs show the change in SLEDAI versus the change in the GSVA enrichment scores for 0 to 12 weeks (top) and 12 to 24 weeks (bottom) **(a)** MS-IFNB1, (**b**) HepC-IFNA2, (**c)** IFNA2, (**d**) IFNB1, (**e**) and IFNW1.

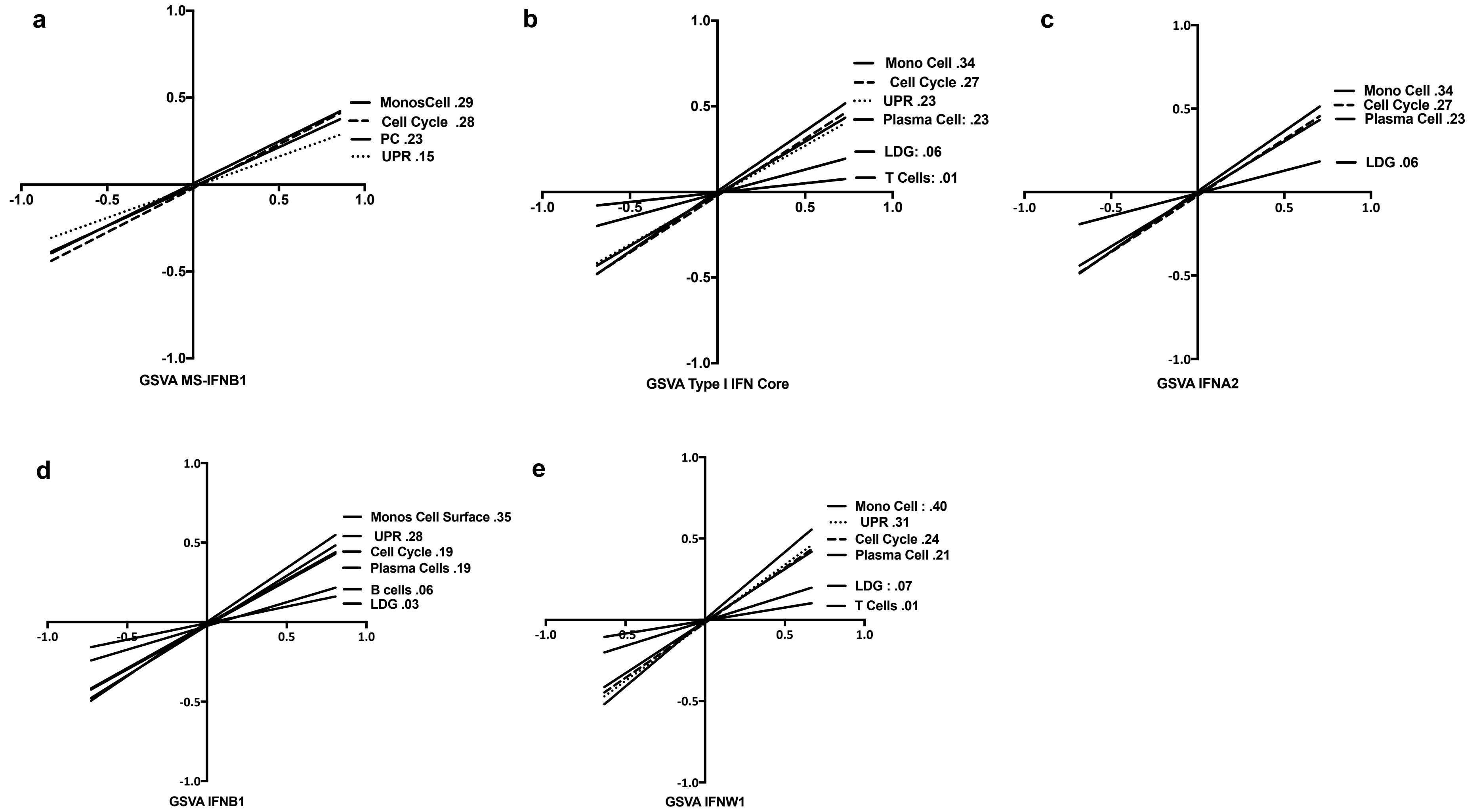

Supplementary Figure 8. IFN Subtypes Are Most Related to Monocyte Cell Surface Transcripts by Linear Regression Analysis. Linear regression analysis between the cell type specific, nonoverlapping IFN signatures and the GSVA enrichment cell type score (y-axis) for the patients from 10 SLE WB and PBMC datasets (**Supplementary Data 19 – 28**). Cell types or signatures significantly ($p < .05$) related to the nonoverlapping IFN score for **(a)** MS-IFNB1, (**b**) type I IFN core, **(c)** IFNA2, (**d**) IFNB1, and **(e)** IFNW1 in at least half of the datasets which had determinable GSVA scores were used to determine overall regression lines for all 10 datasets. r^2 values are listed after the GSVA enrichment category. PC (plasma cell), UPR (unfolded protein response), LDG (low density granulocyte),

Supplementary Figure References

1. Waddell, S.J. et al. Interferon-induced transcriptional programs in human peripheral blood cells. *PLoS One* **5**(3): e9753(2010).
2. Chiche, L. et al. Modular transcriptional repertoire analyses of adults with systemic lupus erythematosus reveal distinct type I and type II interferon signatures. *Arthritis Rheumatol*. **66**(6):1583-95(2014).
3. de Weerd, N.A. et al. Structural basis of a unique interferon-beta signaling axis mediated via the receptor IFNAR1. *Nat. Immunol*. **14**, 901-907(2013).
