# Supplementary material for: Gene expression analysis delineates the potential roles of multiple interferons in systemic lupus erythematosus
Source: Commun Biol. 2019 Apr 23;2:140. doi: 10.1038/s42003-019-0382-x (PMC6478921; doi:10.1038/s42003-019-0382-x)
Supplement: Supplementary file 2 — Description of additional supplementary items [file 42003_2019_382_MOESM2_ESM.docx]

**Description of Additional Supplementary Items**

**Title of file: Supplementary Data**

**Format: .xlsx**

**Overview: 32 Supplementary Data files referred to in the manuscripts as Supplementary Data 1 - 32 in 32 sequential excel sheet tabs and Supplementary Data References**

- Supplementary Data 1. Description: SLE Datasets
- Supplementary Data 2. Description: Induced Transcripts in PBMC by Waddell et al. (2010).
- Supplementary Data 3. Description: Interferome V2.01. of Induced Transcripts Listed for MS-IFNB1 and HepC IFNA2
- Supplementary Data 4. Description: Two-way ANOVA Discriminate Analysis of IFN Signatures
- Supplementary Data 5. Description: Z Score Calculations for SLE WB, PBMC, Tissues and Controls
- Supplementary Data 6. Description: Active (SLEDAI ≥ 6) and Inactive (SLEDAI < 6) SLE Patients
- Supplementary Data 7. Description: IGS Positive and Negative SLE Patients
- Supplementary Data 8. Description: WGCNA Constructed IGS Modules and Correlation (Pearson's r) to SLEDAI.
- Supplementary Data 9. Description: Standard Deviation (SD) Calculations for GSE88885 Time Course Dataset
- Supplementary Data 10. Description: Standard Deviation (SD) Calculations for GSE88886 Time Course Dataset
- Supplementary Data 11. Description: Standard Deviation (SD) Calculations for GSE72747 Time Course Dataset
- Supplementary Data 12. Description: Gene Symbols for GSVA Enrichment Cell Type and Functional Categories
- Supplementary Data 13. Description: Overlap of GSVA Enrichment Category Gene Symbols with SLE Datasets
- Supplementary Data 14. Description: r^2^ Values for GSVA Enrichment Cell Type Categories with Linear Regression P values less than or equal to .05
- Supplementary Data 15. Description: Five WB and Two PBMC Linear Regression for SLEDAI versus Hematopoietic Cell GSVA Enrichment Score
- Supplementary Data 16. Description: Nonoverlapping IFN Signatures for Linear Regression Analysis to Cell and Process Modules
- Supplementary Data 17. Description: Linear Regression Analysis Using Nonoverlapping HepC-IFNA2 signature and Cell Type GSVA Enrichment Scores
- Supplementary Data 18. Description: Linear Regression Analysis Using Nonoverlapping HepC-IFNA2 signature and Cell Type GSVA Enrichment Scores from 10 SLE Datasets combined
- Supplementary Data 19. Description: Linear Regression Analysis Using Nonoverlapping MS-IFNB1 signature and Cell Type GSVA Enrichment Scores
- Supplementary Data 20. Description: Linear Regression Analysis Using Nonoverlapping MS-IFNB1 signature and Cell Type GSVA Enrichment Scores from 10 SLE Datasets combined
- Supplementary Data 21. Description: Linear Regression Analysis Using Nonoverlapping PBMC-IFNA2 signature and Cell Type GSVA Enrichment Scores
- Supplementary Data 22. Description: Linear Regression Analysis Using Nonoverlapping IFNA2 signature and Cell Type GSVA Enrichment Scores from 10 SLE Datasets combined
- Supplementary Data 23. Description: Linear Regression Analysis Using Nonoverlapping PBMC- IFNB1 signature and Cell Type GSVA Enrichment Scores
- Supplementary Data 24. Description: Linear Regression Analysis Using Nonoverlapping IFNB1 signature and Cell Type GSVA Enrichment Scores from 10 SLE Datasets combined
- Supplementary Data 25. Description: Linear Regression Analysis Using Nonoverlapping IFNW1 signature and Cell Type GSVA Enrichment Scores
- Supplementary Data 26. Description: Linear Regression Analysis Using Nonoverlapping IFNW1 signature and Cell Type GSVA Enrichment Scores from 10 SLE Datasets combined
- Supplementary Data 27. Description: Linear Regression Analysis Using Nonoverlapping IFN Core signature and Cell Type GSVA Enrichment Scores
- Supplementary Data 28. Description: Linear Regression Analysis Using Nonoverlapping IFN Core signature and Cell Type GSVA Enrichment Scores from 10 SLE Datasets combined
- Supplementary Data 29. Description: Plasmacytoid Dendritic Cell Specific Transcripts in SLE PBMC and WB
- Supplementary Data 30. Description: IFN Core Signature Transcript Expression for DE Analysis of Monocytes, T cells and B cells to each other
- Supplementary Data 31. Description: Transcripts Not DE between T Cells, B Cells and Monocytes; Limma DE Analysis of SLE Purified T Cells, B Cells and Monocytes to Healthy Controls
- Supplementary Data 32. Description: WGCNA IFN Modules from SLE Monocytes, T Cells and B Cells
- Supplementary Data References
