# Supplementary material for: Gene expression analysis delineates the potential roles of multiple interferons in systemic lupus erythematosus
Source: Commun Biol. 2019 Apr 23;2:140. doi: 10.1038/s42003-019-0382-x (PMC6478921; doi:10.1038/s42003-019-0382-x)
Supplement: Supplementary file 3 — Reporting Summary [file 42003_2019_382_MOESM3_ESM.pdf]

nature research

Corresponding author(s): Catalina, MD

Last updated by author(s): March 1, 2019

Reporting Summary

Nature Research wishes to improve the reproducibility of the work that we publish. This form provides structure for consistency and transparency in reporting. For further information on Nature Research policies, see Authors & Referees and the Editorial Policy Checklist.

Statistics

For all statistical analyses, confirm that the following items are present in the figure legend, table legend, main text, or Methods section.

n/a	Confirmed	
☐	☒	The exact sample size (n) for each experimental group/condition, given as a discrete number and unit of measurement
☐	☒	A statement on whether measurements were taken from distinct samples or whether the same sample was measured repeatedly
☐	☒	The statistical test(s) used AND whether they are one- or two-sided<br>*Only common tests should be described solely by name; describe more complex techniques in the Methods section.*
☐	☒	A description of all covariates tested
☐	☒	A description of any assumptions or corrections, such as tests of normality and adjustment for multiple comparisons
☐	☒	A full description of the statistical parameters including central tendency (e.g. means) or other basic estimates (e.g. regression coefficient) AND variation (e.g. standard deviation) or associated estimates of uncertainty (e.g. confidence intervals)
☐	☒	For null hypothesis testing, the test statistic (e.g. F, t, r) with confidence intervals, effect sizes, degrees of freedom and P value noted<br>*Give P values as exact values whenever suitable.*
☒	☐	For Bayesian analysis, information on the choice of priors and Markov chain Monte Carlo settings
☒	☐	For hierarchical and complex designs, identification of the appropriate level for tests and full reporting of outcomes
☐	☒	Estimates of effect sizes (e.g. Cohen's d, Pearson's r), indicating how they were calculated

Our web collection on statistics for biologists contains articles on many of the points above.

Software and code

Policy information about availability of computer code

Data collection	N/A
Data analysis	Freely available, open source code was used for these analyses. Code examples for LIMMA, GSVA and WGCNA used in this paper are available at figshare, www.figshare.com. File names are AMPEL BioSolutions LIMMA DIfferential Expression Analysis Code, AMPEL BioSolutions Gene Set Variation Analysis Code, and AMPEL BioSolutions Weighted Correlation Network Analysis WGCNA Code. LIMMA for R is available at https://www.bioconductor.org/packages/release/bioc/html/limma.html. GSVA for R is available at www.bioconductor.org/packages/release/bioc/html/GSVA.html. WGCNA for R is available at https://horvath.genetics.ucla.edu/html/CoexpressionNetwork/Rpackages/WGCNA/.

For manuscripts utilizing custom algorithms or software that are central to the research but not yet described in published literature, software must be made available to editors/reviewers. We strongly encourage code deposition in a community repository (e.g. GitHub). See the Nature Research guidelines for submitting code & software for further information.

Data

Policy information about availability of data

All manuscripts must include a data availability statement. This statement should provide the following information, where applicable:
- Accession codes, unique identifiers, or web links for publicly available datasets
- A list of figures that have associated raw data
- A description of any restrictions on data availability

All microarray datasets in this publication are available on the NCBI's database Gene Expression Omnibus (GEO) (https://www.ncbi.nlm.nih.gov/geo/) except the FDA PBMC dataset. The FDA PBMC microarray dataset is available for download in the Supplementary Materials, File S1 of Labonte , A.C. Identification of alterations in macrophage activation associated with disease activity in systemic lupus erythematosus. PLoS One. 13(12):e0208132 (2018). Accession codes for the datasets are given in Supplementary Table 1 and throughout the manuscript.

Field-specific reporting

Please select the one below that is the best fit for your research. If you are not sure, read the appropriate sections before making your selection.

☒ Life sciences ☐ Behavioural & social sciences ☐ Ecological, evolutionary & environmental sciences

For a reference copy of the document with all sections, see nature.com/documents/nr-reporting-summary-flat.pdf

Life sciences study design

All studies must disclose on these points even when the disclosure is negative.

Sample size	21 systemic lupus erythematosus (SLE) patient and control microarray datasets comprising more than 2000 SLE patients were used for these analyses.
Data exclusions	There were no data exclusions.
Replication	A metaanalysis approach was performed across 7 SLE datasets to determine relationships to the disease measure systemic lupus erythematosus disease activity index (SLEDAI), and across 10 SLE datasets to understand the IFN signature.
Randomization	This meta-analysis approach did not require randomization. Patients and controls for each dataset were previously determined and used appropriately.
Blinding	Blinding was not relevant to this study.

Reporting for specific materials, systems and methods

We require information from authors about some types of materials, experimental systems and methods used in many studies. Here, indicate whether each material, system or method listed is relevant to your study. If you are not sure if a list item applies to your research, read the appropriate section before selecting a response.

Materials & experimental systems

n/a	Involved in the study	
☒	☐	Antibodies
☒	☐	Eukaryotic cell lines
☒	☐	Palaeontology
☒	☐	Animals and other organisms
☒	☐	Human research participants
☒	☐	Clinical data

Methods

n/a	Involved in the study	
☐	☒	ChIP-seq
☒	☐	Flow cytometry
☒	☐	MRI-based neuroimaging

ChIP-seq

Data deposition

☒ Confirm that both raw and final processed data have been deposited in a public database such as GEO.

☐ Confirm that you have deposited or provided access to graph files (e.g. BED files) for the called peaks.

Data access links
May remain private before publication.

GSE88884 - https://www.ncbi.nlm.nih.gov/geo/query/acc.cgi?acc=GSE88884;
GSE45291 - https://www.ncbi.nlm.nih.gov/geo/query/acc.cgi?acc=GSE45291;
GSE22098 - https://www.ncbi.nlm.nih.gov/geo/query/acc.cgi?acc=GSE22098;
GSE61635 - https://www.ncbi.nlm.nih.gov/geo/query/acc.cgi?acc=GSE61635;
GSE29536 - https://www.ncbi.nlm.nih.gov/geo/query/acc.cgi?acc=GSE29536;
GSE39088 - https://www.ncbi.nlm.nih.gov/geo/query/acc.cgi?acc=GSE39088;
GSE49454 - https://www.ncbi.nlm.nih.gov/geo/query/acc.cgi?acc=GSE49454;
GSE50772 - https://www.ncbi.nlm.nih.gov/geo/query/acc.cgi?acc=GSE50772;
GSE38351 - https://www.ncbi.nlm.nih.gov/geo/query/acc.cgi?acc=GSE38351;
GSE10325 - https://www.ncbi.nlm.nih.gov/geo/query/acc.cgi?acc=GSE10325;
GSE52471 - https://www.ncbi.nlm.nih.gov/geo/query/acc.cgi?acc=GSE52471;
GSE72535 - https://www.ncbi.nlm.nih.gov/geo/query/acc.cgi?acc=GSE72535;
GSE36700 - https://www.ncbi.nlm.nih.gov/geo/query/acc.cgi?acc=GSE36700;
GSE32591 - https://www.ncbi.nlm.nih.gov/geo/query/acc.cgi?acc=GSE32591;
GSE72747 - https://www.ncbi.nlm.nih.gov/geo/query/acc.cgi?acc=GSE72747;
GSE88885 - https://www.ncbi.nlm.nih.gov/geo/query/acc.cgi?acc=GSE88885;
GSE88886 - https://www.ncbi.nlm.nih.gov/geo/query/acc.cgi?acc=GSE88886;

	FDA PBMC - available File S1 https://journals.plos.org/plosone/article?id=10.1371/journal.pone.0208132#sec022
Files in database submission	There are no files being submitted to any database for this paper.
Genome browser session (e.g. UCSC)	There were no genome browser sessions. The Interferome database was used to define genes previously described as being increased in response to type or type II interferons.

Methodology

Replicates	A meta-analysis approach was performed across 7 SLE datasets to determine relationships to the disease SLEDAI and across 10 SLE datasets to understand the IFN signature.
Sequencing depth	This data was taken from the GEO repository
Antibodies	No antibodies were used in this study.
Peak calling parameters	This data was taken from the publicly available GEO repository. This information may be available in the initial publications of the datasets (if they have a published paper associated with them) but likely is not available.
Data quality	This data was taken from the publicly available GEO repository. This information may be available in the initial publications of the datasets (if they have a published paper associated with them) but likely is not available.
Software	No new software was developed for this study. Publicly available data and software was used for these analyses.
